# An Enhanced Genomic Transformer for Multi-Dataset Forward-Validation Benchmarking in Pig Genomic Prediction

**DOI:** 10.3390/ani16203284

**Published:** 2026-10-18

**Authors:** Weixi Xiang, Zhaoxin Li, Qixin Sun, Xiujuan Chai, Tan Sun

**Affiliations:** 1 Agricultural Information Institute, Chinese Academy of Agricultural Sciences, Beijing 100081, China; 2 Key Laboratory of Agricultural Big Data, Ministry of Agriculture and Rural Affairs, Beijing 100081, China

**Keywords:** genomic prediction, pig breeding, forward validation, transformer, multi-task learning

## Abstract

Genomic prediction models in pig breeding should forecast the performance of future selection candidates, yet random cross-validation does not accurately reflect this sequential setting. Here, we introduce an enhanced genomic transformer (EGT) that compresses genome-wide SNP profiles into compact tokens and incorporates a differentiable scoring term alongside mean squared error (MSE) during training. We constructed 70%/10%/20% forward-validation datasets for three pig datasets and evaluated single-trait and multi-task EGT variants against GBLUP, support-vector regression, random forest, XGBoost, CNN, and RNN across 12 prediction tasks. The better-performing EGT variant achieved a higher test-set Pearson correlation coefficient (PCC) than the baseline for 10 of the 12 prediction tasks, with the largest improvement on LDL-C (+0.057). In BloodLipid ablations, autoencoder-only representation yielded the highest mean PCC (0.285), while Pearson–MSE objective reached 0.293 versus 0.291 for MSE-only training. Integrated-gradient analysis revealed a recurrent candidate region on *Sus scrofa* chromosome 13 across three lipid traits, and masking the top-attributed features reduced predictive performance more than a marker-count-matched random control for HDL-C, LDL-C and TG, whereas masking increased test-set PCC for TCHOL. This work establishes forward-validation benchmarks across three pig datasets and reports results to inform model selection and region prioritization in pig breeding.

## Simple Summary

Genomic prediction can help pig breeders identify promising animals before all performance traits are measured. Many studies randomly divide animals into training and testing groups, but this may not reflect breeding practice, where animals born or evaluated later must be predicted from earlier information. We compared statistical, machine learning, and deep learning approaches using three publicly available pig datasets containing production, growth, feed-efficiency, and blood-lipid traits. We developed a model that summarizes thousands of genetic markers into a small number of units before combining information across the genome. Across 12 traits, the strongest version of this model performed better than the best conventional method for 10 of them, with the largest improvement on LDL-C; on one trait a tree-based method was clearly better, and on two the two approaches were indistinguishable. The predictions also span a narrower range of values than the observed phenotypes, so the numbers need to be recalibrated before they are used to compare animals whose phenotypes differ in reliability. Additional experiments compared alternative ways of representing genetic markers and training the model. We also examined which genomic regions contributed to its predictions by ranking markers and temporarily masking selected regions. The results provide a reproducible framework for evaluating genomic prediction models under a practical forward-looking design and for prioritizing genomic regions for further study.

## 1. Introduction

Genomic selection uses dense marker information to evaluate animals before late, expensive, or sex-limited phenotypes are observed, thereby supporting shorter generation intervals and more efficient livestock improvement [1,2,3,4]. In pig breeding, production, survival, and maternal traits are predicted from a finite reference population and then used to inform decisions about animals outside that fitted reference set [5,6]. The practical question is therefore not only whether a model fits the reference population, but whether it can accurately predict phenotypes for candidate animals evaluated later.

Notably, our forward-validation benchmark was built upon a unified reorganization of existing public pig datasets, with dataset-specific rules applied to ensure comparability across heterogeneous traits. The ordering criteria were as follows: pedigree-generation depth for PIC [5], birth year for PigHeaT [7] (2013 for training and validation, 2014 for testing), and experimental batch for each BloodLipid population [8]; the resulting order subsequently partitioned the forward-validation training, validation, and test sets.

The choice of prediction model remains an open question in a benchmark that spans multiple pig datasets and trait types [9]. GBLUP provides an established linear reference [10,11,12,13], whereas support-vector regression (SVR), random forest (RF), extreme gradient boosting (XGBoost), convolutional neural networks (CNNs), and recurrent neural networks (RNNs) provide commonly used alternatives for genomic prediction. Recent pig studies have applied CNN and residual-attention architectures, but their performance varies across traits and evaluation metrics [14,15,16]. These methods represent SNP information in different ways: RF and XGBoost model nonlinear effects, CNNs summarize local patterns [17,18,19], and RNNs model ordered SNP sequences. Evaluating these models on identical data partitions allows a direct comparison among established statistical, machine learning, and deep learning alternatives [20].

Transformer models use self-attention to exchange information among different parts of the SNP representation, which may help integrate genomic signals distributed across the genome [21,22,23,24]. Recent studies have applied attention-based genomic prediction models to pig growth traits and to other genomic prediction datasets [25,26,27]. Differences in input representation, trait composition, and validation design among these studies make a common evaluation on the same data partitions valuable. In this study, we introduce the enhanced genomic transformer (EGT), a genomic prediction model that tokenizes genome-wide SNP genotypes into compact representations prior to self-attention. We evaluate single-trait and multi-task EGT against six established methods on the same forward-validation training, validation, and test sets in the constructed multi-dataset benchmark.

Beyond model architecture, the choice of objective loss function for training EGT may also critically affect phenotype prediction performance. Mean squared error (MSE) penalizes absolute prediction error and is a standard objective for phenotype prediction. Because genomic prediction is used to rank candidate animals for selection, we also examine objectives that encourage agreement between observed and predicted phenotypes across animals [28]. Pearson correlation, Spearman correlation, and $R^{2}$ describe complementary aspects of prediction: Pearson correlation measures linear agreement, Spearman correlation measures rank agreement, and $R^{2}$ measures goodness of fit. The training objective combines MSE with one of the score terms to assess whether the score term improves prediction performance while MSE controls absolute prediction error.

**Figure 1:**
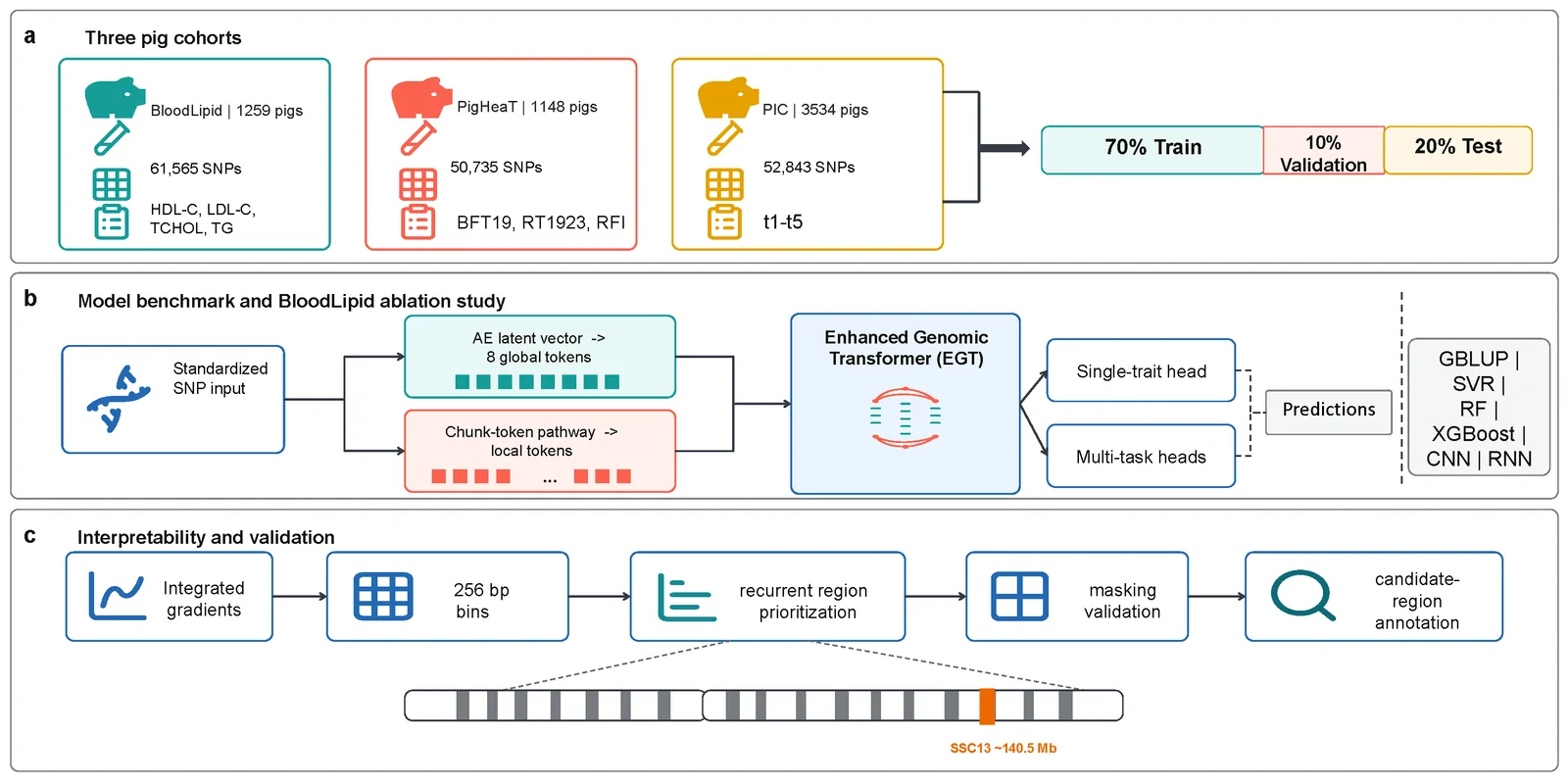
Forward-validation workflow. For forward validation, animals were arranged from earlier to later using the temporal ordering information available in each dataset: birth year for PigHeaT (2013 for training and validation, 2014 for testing), pedigree-generation depth for PIC, and experimental batch within each BloodLipid population. The ordered animals are then divided into 70% training, 10% validation, and 20% test sets for forward validation. Six baseline models, single-trait EGT, and multi-task EGT (MTL-EGT) are compared across 12 prediction tasks. For the four directly measured BloodLipid traits, ablation analyses compare AE-only, chunk-only, and fused global–local representations, single-trait and multi-task learning, model capacity, and MSE combined with Pearson, Spearman, or $R^{2}$ score terms. Integrated gradients and masking are then used to identify genomic regions that contribute to EGT predictions, and the attributions are aggregated by genomic coordinate into half-open 256-bp bins.

The overall workflow of the study is illustrated in Figure 1. The three contributions of this study are as follows.

(1) We establish a forward-validation benchmark across three public pig datasets and 12 prediction tasks.
(2) We develop the enhanced genomic transformer (EGT) for pig genomic prediction. EGT compresses genome-wide SNP genotypes into compact tokens before self-attention and combines MSE with a differentiable correlation term during training.
(3) For the BloodLipid dataset, we use integrated gradients and masking analyses to assess the biological interpretability of EGT predictions and to prioritize genomic regions contributing to those predictions.

## 2. Materials and Methods

### 2.1. Datasets and Forward-Validation Design

Three publicly available pig datasets were analyzed. The PIC dataset included 3534 genotyped animals, five anonymous production traits (t1–t5), and 52,843 single-nucleotide polymorphisms (SNPs) after preprocessing [5]. The PigHeaT dataset included 1148 genotyped animals and three traits: ultrasonic backfat thickness at week 19 (BFT19), the mean rectal temperature at weeks 19 and 23 measured under heat-stress challenge (RT1923), and residual feed intake (RFI). Quality control retained 50,735 of 60,466 de-duplicated autosomal markers at a call rate of at least 0.95 and a minor allele frequency of at least 0.01 [7]. The BloodLipid dataset included 1259 animals from DLY, EHL, and Laiwu populations, 61,565 SNPs, and serum lipid measurements [8]. We analyzed four directly measured lipid traits: high-density lipoprotein cholesterol (HDL-C), low-density lipoprotein cholesterol (LDL-C), total cholesterol (TCHOL), and triglycerides (TG). The atherogenic index and the HDL-C/LDL-C ratio were excluded because they are deterministic combinations of these measurements rather than independent phenotypes. The marker sets of PIC and BloodLipid are used as released by their repositories, and the numbers of markers that a minor-allele-frequency and call-rate mask would additionally remove from each dataset are reported in Table S3.

All marker positions in this study refer to the Sscrofa10.2 assembly (GCA_000003025.4). The marker map derives from the Illumina PorcineSNP60 array (Illumina, Inc., San Diego, CA, USA) and contains 61,565 markers after de-duplication.

Each dataset was divided into training, validation, and test sets at a 7:1:2 ratio for forward validation, according to temporal ordering information available in the released data. PIC animals were sorted by pedigree-generation depth inferred from the available parent links. PigHeaT animals were sorted by birth year: animals born in 2013 formed the training and validation sets (804 and 122 animals) and animals born in 2014 formed the test set (222 animals). BloodLipid animals were sorted by experimental batch within each population. These orderings were used to assign the training, validation, and test sets for forward validation. Missing marker genotype values were imputed using marker means calculated from the training set. Optimisation settings for every model, including the differences between EGT and the CNN and RNN baselines, are reported in Table S14. Marker scaling, phenotype scaling, and all corresponding transformations were estimated from the training set and applied unchanged to the validation and test sets. Marker genotypes were scaled to zero mean and unit variance with the training-set marker mean and standard deviation. Phenotypes were scaled in the same way, $z_{i}=\left(y_{i}-{\bar{y}}_{\mathrm{train}}\right)/{\mathrm{SD}}_{\mathrm{train}}$, and models were trained on *z*; every reported statistic was computed after the inverse transformation ${\hat{y}}_{i}={\hat{z}}_{i}\;{\mathrm{SD}}_{\mathrm{train}}+{\bar{y}}_{\mathrm{train}}$, so predictions, bias and regression slopes are all expressed on the original phenotypic scale, and the training-set phenotypic standard deviations are reported in Table S4. Marker genotypes were scaled to zero mean and unit variance with the training-set marker mean and standard deviation. Phenotypes were scaled in the same way, $z_{i}=\left(y_{i}-{\bar{y}}_{\mathrm{train}}\right)/{\mathrm{SD}}_{\mathrm{train}}$, and models were trained on *z*; every reported statistic was computed after the inverse transformation ${\hat{y}}_{i}={\hat{z}}_{i}\;{\mathrm{SD}}_{\mathrm{train}}+{\bar{y}}_{\mathrm{train}}$, so predictions, bias and regression slopes are all expressed on the original phenotypic scale, and the training-set phenotypic standard deviations are reported in Table S4.

### 2.2. Enhanced Genomic Transformer

**Figure 2:**
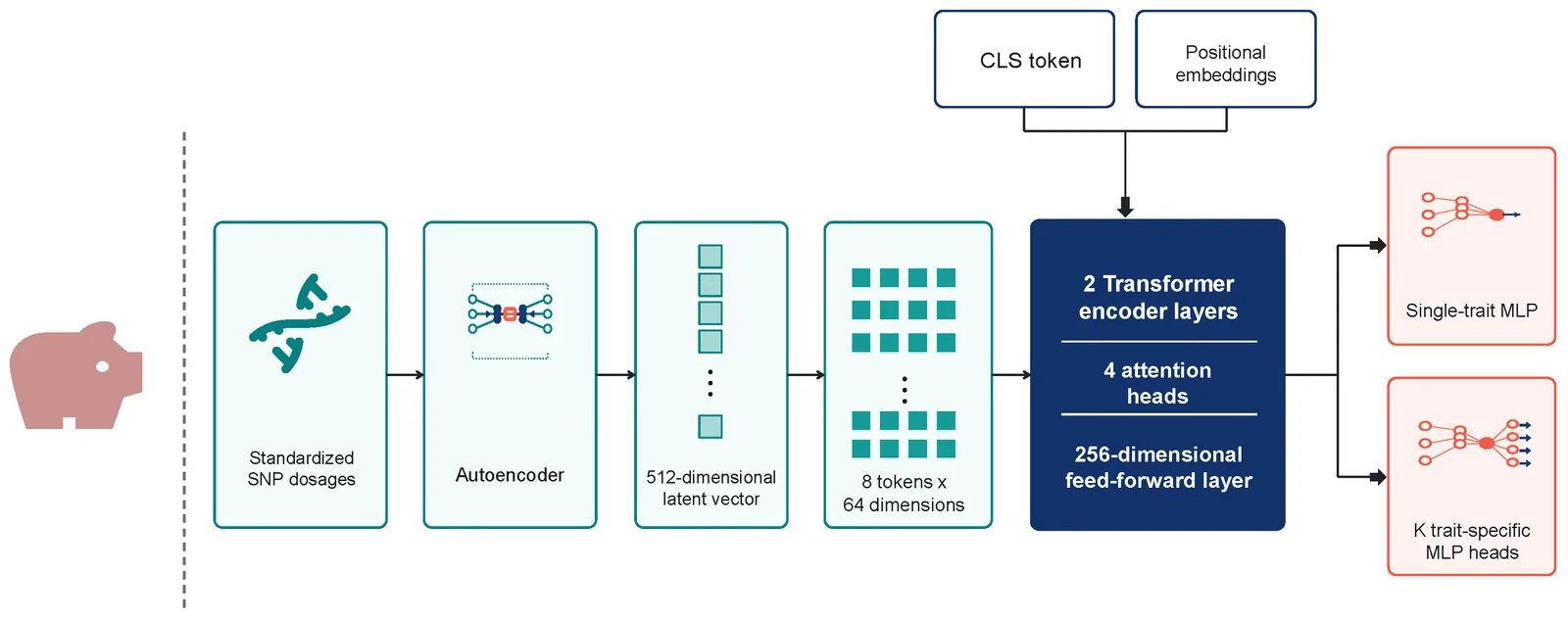
Architecture of the enhanced genomic transformer (EGT). Standardized SNP genotype values are compressed into a 512-dimensional autoencoder latent vector and reshaped into eight 64-dimensional tokens. A classification token and positional embeddings enter two Transformer encoder layers with four attention heads and a 256-dimensional feed-forward block. The pooled representation is passed to either one single-trait prediction head or multiple trait-specific heads connected to a shared encoder.

**Figure 3:**
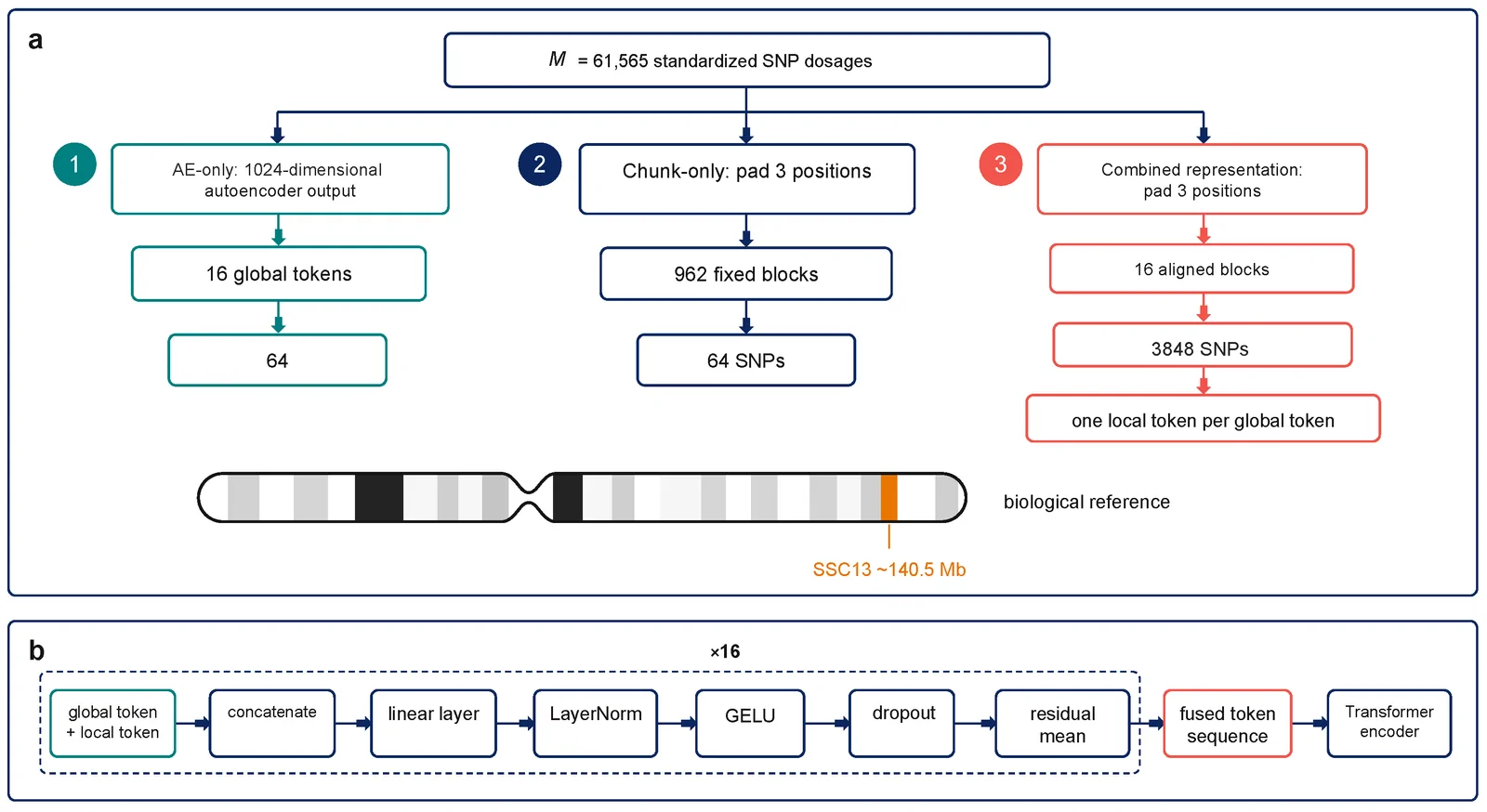
Variable EGT architectures. (**a**) The three input representations, AE-only, chunk-only and the combined representation, together with the number of tokens and markers obtained by each. (**b**) The fusion block, applied 16 times, which merges an aligned global token and a local token into one fused token before the Transformer encoder. AE-only forms global tokens from an autoencoder output, chunk-only forms local tokens from consecutive SNP blocks, and the fused representation combines aligned global and local tokens before Transformer encoding. The representation and task-formulation comparisons used a common 16-token/64-dimensional EGT backbone. The capacity comparison included the 8-token/64-dimensional configuration used in the main analyses and the 16/64, 16/96, and 32/96 alternatives.

Genomic prediction commonly relies on genome-wide SNP profiles containing tens of thousands of markers. When every marker is treated as a separate input, self-attention must operate over a long marker sequence. EGT addresses this input representation by compressing the genome-wide SNP profile into a compact token sequence before Transformer encoding. As shown in Figure 2, standardized SNP genotype values were compressed by an autoencoder with a 1024-unit hidden layer and a 512-dimensional latent layer [29]. The autoencoder was fitted on the training set only: its weights were estimated from training genotypes alone, and the marker means used for imputation and scaling were likewise training-set quantities, so no validation or test genotype enters the representation. The latent vector was reshaped into eight tokens with 64 features each. A learnable classification token and learned positional embeddings were added before two Transformer encoder layers with four attention heads and a feed-forward width of 256. Each encoder layer used residual connections, layer normalization, Gaussian error linear unit activations, and dropout. The pooled classification-token representation was passed through a 128-unit hidden prediction head. Single-trait EGT produced one phenotype prediction for a trait, whereas MTL-EGT shared the encoder within a prespecified dataset-level trait group and retained a separate output head for each trait. As illustrated in Figure 3, the genome-wide SNP input was processed in three ways: AE-only formed global tokens from the autoencoder output, chunk-only formed local tokens from consecutive SNP blocks, and fused combined aligned global and local tokens before Transformer encoding. The representation and task-formulation comparisons used a common 16-token/64-dimensional EGT backbone, whereas the capacity comparison evaluated the 8-token/64-dimensional main configuration together with 16/64, 16/96, and 32/96 alternatives.

### 2.3. Benchmark Models and Training Configurations

We compared GBLUP, linear support-vector regression (SVR), random forest (RF), extreme gradient boosting (XGBoost), convolutional neural networks (CNNs), recurrent neural networks (RNNs), single-trait EGT, and multi-task EGT (MTL-EGT). GBLUP was included as a relationship-based genomic prediction model using a genomic relationship matrix derived from SNP markers [1,2,30,31]. GBLUP used the method-1 genomic relationship matrix of VanRaden [30], computed from marker genotypes centered on twice the training-set allele frequency, with missing genotypes imputed to the training marker mean and the genomic relationship scaled by twice the sum of p(1−p) over markers. Linear SVR, RF, and XGBoost were included as machine learning models based on linear prediction, bagged decision trees, and boosted decision trees, respectively [20,32,33,34]. CNNs and RNNs were included as deep learning models that learn convolutional patterns and sequential representations from ordered SNP inputs, respectively. Single-trait EGT fitted one model for each trait, whereas MTL-EGT used a shared encoder with separate output heads for traits within the same dataset [35].

GBLUP, SVR, RF, XGBoost, CNN, RNN, and single-trait EGT were fitted separately for each trait using the forward-validation training sets. MTL-EGT was trained jointly for traits within the same dataset, with a shared encoder and a separate prediction head for each trait. The CNN processed each ordered SNP vector as a one-dimensional signal, whereas the RNN processed consecutive SNP blocks as an ordered sequence. Hyperparameters, loss weights and checkpoints were tuned on the validation dataset with a frozen selection seed of 42, before the test datasets were evaluated; the tuning runs contain validation metrics only. The comparison spans eight methods, and each of them was trained for every trait so that all eight are evaluated under the same data partitions. The reported result compares them at their best under one and the same rule, EGT with the better of its two variants and the reference with the better of the six conventional models. Table S15 repeats the comparison under a second rule in which each side is taken at its best on the validation dataset, so that no test-set performance enters the choice of which model is reported, and gives the validation PCC of all eight models in Table S16. The optimisation settings of every model are listed in Table S14, and the selected values are the ones used for every reported result. Hyperparameters were tuned on the validation dataset for every model, the baselines included, with the test datasets left untouched. The tuning effort is therefore not identical across models, and the difference is one of range rather than of principle; part of any gap may reflect that difference rather than the representations themselves, which is an inherent limitation of the comparison and should be read with the reported differences in mind. Analyses used Python 3.10.15, PyTorch 2.9.1, NumPy 1.26.4, pandas 2.3.3, SciPy 1.15.3, scikit-learn 1.7.2 and XGBoost 3.2.0, with figures prepared in Matplotlib 3.10.8; training and inference ran on a workstation with an AMD Ryzen Threadripper PRO 7975WX CPU (Advanced Micro Devices, Inc., Santa Clara, CA, USA) and an NVIDIA RTX A6000 GPU (NVIDIA Corporation, Santa Clara, CA, USA).

### 2.4. MSE and Differentiable Score Objective

Genomic selection requires both a useful ordering of candidate animals and predictions on the observed phenotype scale. A correlation score is informative for the first requirement but is unchanged by a positive shift or rescaling of the predictions. We therefore combined a differentiable score term with MSE, which constrains predictions to remain within the range of observed phenotypic values; we subsequently compared these combined objectives. For a mini-batch containing *K* modeled traits, the EGT training objective was defined as:

(1) $$L=\alpha \frac{1}{K}\sum_{k=1}^{K}w_{k}\;\mathrm{MSE}\left(y_{k},{\hat{y}}_{k}\right)-\left(1-\alpha \right)\frac{1}{K}\sum_{k=1}^{K}w_{k}\;s\left(y_{k},{\hat{y}}_{k}\right),$$

Here, $y_{k}$ and ${\hat{y}}_{k}$ are the observed and predicted phenotype values, *s* is a differentiable score, and 0≤α≤1 is the MSE weight. A missing-label mask restricted each trait term to animals with observed phenotypes. For single-trait training, K=1, $w_{k}=1$. For multi-task training, the number of observed labels in the training set, $n_{k}$, defined $w_{k}=n_{k}^{-0.5}/{\mathrm{mean}}_{j}\left(n_{j}^{-0.5}\right)$, which normalized the average trait weight to one. Thus, α=1 gives the MSE-only endpoint, α=0 gives the score-only endpoint, and intermediate values combine the MSE and score terms.

### 2.5. Experimental Setup for Biological Interpretability

We conducted biological interpretability analyses for LDL-C, TCHOL, and TG. For each test animal, integrated gradients were calculated using 24 interpolation steps from an all-zero vector in standardized input space [36,37]. Marker genotype values were standardized using marker-specific means and scaling parameters estimated from the training set. Consequently, a standardized value of zero for a marker corresponds to a genotype encoding equal to its training-set mean, and the all-zero vector serves as a mean-genotype baseline. It thus encodes no deviation information from that baseline in the model input; it does not imply that the corresponding SNP lacks biological function or information contribution. The interpretability and masking analyses were run on one forward-validation training run of the main EGT configuration per trait, with the loss weight selected on the validation dataset, so the masking effect sizes below come from that run. Absolute attributions were averaged across test animals to obtain SNP-level importance. When chromosome and base-pair positions were available, SNPs were grouped by coordinate into half-open 256-bp bins, [start,end). Because the array markers of the BloodLipid dataset are on average about 40 kb apart, a 256-bp bin is far narrower than the marker spacing and contains at most a single SNP, so the bin-level analyses below operate on individual markers rather than on groups of markers; the bin holding the top-ranked marker contains exactly one. Bin importance was calculated as the sum of the SNP importance values within that bin.

To assess whether highly attributed features affected prediction, we masked the top-ranked 1%, 5% and 10% of bins and compared the result with a random control that masks the same number of markers, using 20 independent random draws per condition and reporting the 2.5 to 97.5 percentile interval of those draws; because a 256-bp bin holds at most one marker at this array density, these fractions are marker fractions as well as bin fractions. For each masking condition, the selected standardized SNP genotype values were set to zero, after which test predictions were recomputed. We additionally masked whole chromosomes and single candidate regions as locus-scale controls: SSC3, which carries the published *APOB* signal for these animals, serves as the positive control, and the SSC13 candidate region as the negative control [38]. Integrated gradients identify highly attributed SNPs and genomic regions in the EGT model, whereas marker-to-gene labels were assigned separately through external positional annotation. The resulting labels provide biological context for model-identified regions and, together with relevant external resources and the literature, support biological interpretation [39,40,41].

### 2.6. Evaluation Metrics

Predictive performance was evaluated using the test-set Pearson correlation coefficient (PCC), root mean squared error (RMSE), and mean absolute error (MAE). RMSE and MAE describe the numerical error of phenotype prediction [42]. For observations $y_{i}$ and predictions ${\hat{y}}_{i}$, PCC was calculated as:

(2) $$r=\frac{\sum_{i}\left(y_{i}-\bar{y}\right)\left({\hat{y}}_{i}-\bar{\hat{y}}\right)}{\sqrt{\sum_{i}{\left(y_{i}-\bar{y}\right)}^{2}}\sqrt{\sum_{i}{\left({\hat{y}}_{i}-\bar{\hat{y}}\right)}^{2}}},$$

RMSE and MAE were calculated as:

(3) $$\mathrm{RMSE}=\sqrt{\frac{1}{n}\sum_{i}{\left(y_{i}-{\hat{y}}_{i}\right)}^{2}},\;\mathrm{MAE}=\frac{1}{n}\sum_{i}\left|y_{i}-{\hat{y}}_{i}\right|.$$

Performance was reported separately for each trait. All metrics were computed on the original phenotypic scale: the standardized predictions were returned to that scale with the training-set mean and standard deviation, ${\hat{y}}_{i}={\hat{z}}_{i}\;{\mathrm{SD}}_{\mathrm{train}}+{\bar{y}}_{\mathrm{train}}$, before PCC, RMSE, MAE, bias and the regression slope of true on predicted values were calculated, so the slope and bias reported below describe the spread of the predictions themselves rather than a standardized rescaling of them. For each trait, gain was calculated as the PCC of the reported EGT variant minus the PCC of the best baseline. The ablation, capacity and loss-weight comparisons are single-run and carry no uncertainty estimate, unlike the replicated benchmark; they share one fixed reference configuration per ablation family, which was executed once in each family and reproduces exactly because the training is deterministic.

## 3. Results

### 3.1. Results of Comparative Experiments

**Figure 4:**
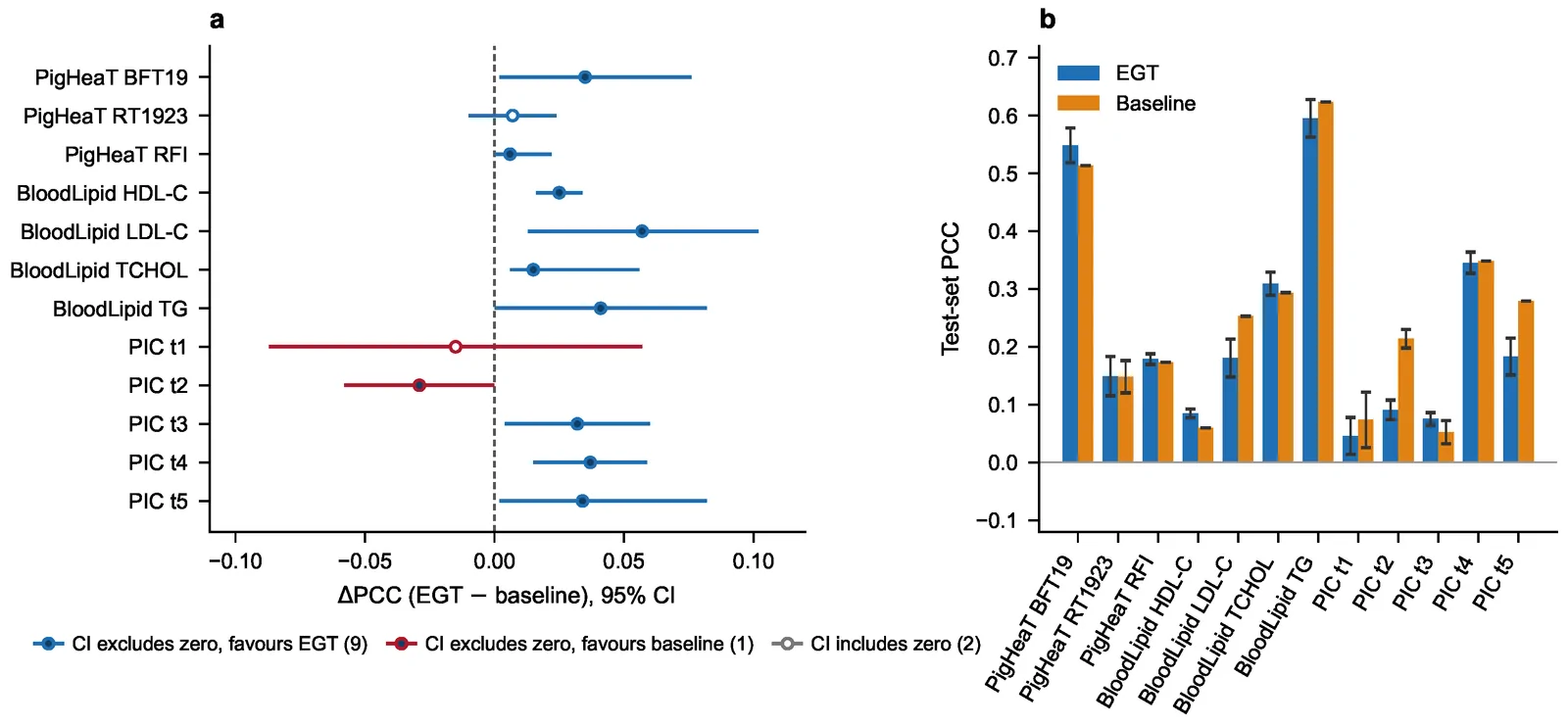
Results of comparative experiments on the forward-validation test sets. (**a**) Difference in test-set PCC between the EGT variant and the baseline of each cell, with its 95% paired-bootstrap confidence interval; filled markers mark the cells whose interval excludes zero. (**b**) Test-set PCC of the EGT variant and of the baseline, with the standard deviation over five replicate seeds. As in Table 2, the EGT variant and the baseline are the best-performing choice of each cell under one and the same rule.

The comparative experiments covered five PIC production traits, three PigHeaT traits and four directly measured BloodLipid traits (Table 1). Every dataset-by-trait-by-model cell was retrained with five independent random seeds, and Table 2 reports the mean and standard deviation over those seeds together with a paired-bootstrap confidence interval on the difference (Figure 4). Compared with the baseline, EGT was numerically higher in 10 of 12 cells and the interval excluded zero in 10 of 12: nine favouring EGT (PigHeaT BFT19 and RFI, all four BloodLipid traits, and PIC t3, t4 and t5) and one favouring the baseline (PIC t2, −0.029 [−0.058, −0.00002]). Only PigHeaT RT1923 and PIC t1 were indistinguishable from zero. The largest improvement was on LDL-C (+0.057 [+0.013, +0.102]). The reported EGT variant and the reported baseline are both the best-performing choice on each test dataset, under one and the same rule, so the two are compared at their best.

Because ranking correlation alone is not sufficient for breeding evaluation, Table 3 reports prediction bias and the regression slope of true phenotype on predicted value for each cell, both computed on the original phenotypic scale. Normalised by the trait’s phenotypic standard deviation, EGT’s absolute bias is smaller than the baseline’s in eight of the twelve cells, with a mean of 0.138 SD against 0.151 SD. The slopes are compressed towards zero for most traits, ranging from 0.092 to 1.259 for EGT, so the predictions rank animals usefully while spanning a narrower range of values than the observed phenotypes.

No baseline model was strongest for every trait. SVR was the best baseline for HDL-C and TCHOL; XGBoost for LDL-C; GBLUP for PigHeaT BFT19 and RFI, TG, and PIC t4 and t5; RF for PigHeaT RT1923 and PIC t2 and t3; and CNN for PIC t1. The best baseline varied across traits, indicating that model choice should be made for each trait. Three cells carry a significant difference and all three favour the baseline: LDL-C, PIC t2 and PIC t5.

### 3.2. Single-Trait and Multi-Task EGT

Both EGT variants were trained for every dataset and trait. The reported variant is the better of the two on each test dataset, which is the convention stated in Table 2.

MTL-EGT uses one shared encoder for traits within the same dataset. It should be most beneficial when jointly modeled traits contain overlapping biological and phenotypic information, because the shared encoder can learn genomic features useful for more than one trait. LDL-C and TCHOL provide a concrete example: LDL-C is a low-density lipoprotein cholesterol fraction that contributes to the total cholesterol measurement (TCHOL). Their overlap in cholesterol metabolism provides shared phenotypic information that can be learned by the common encoder, and MTL-EGT achieved the highest PCC among all compared models for both traits.

### 3.3. BloodLipid Ablation Experiments

**Figure 5:**
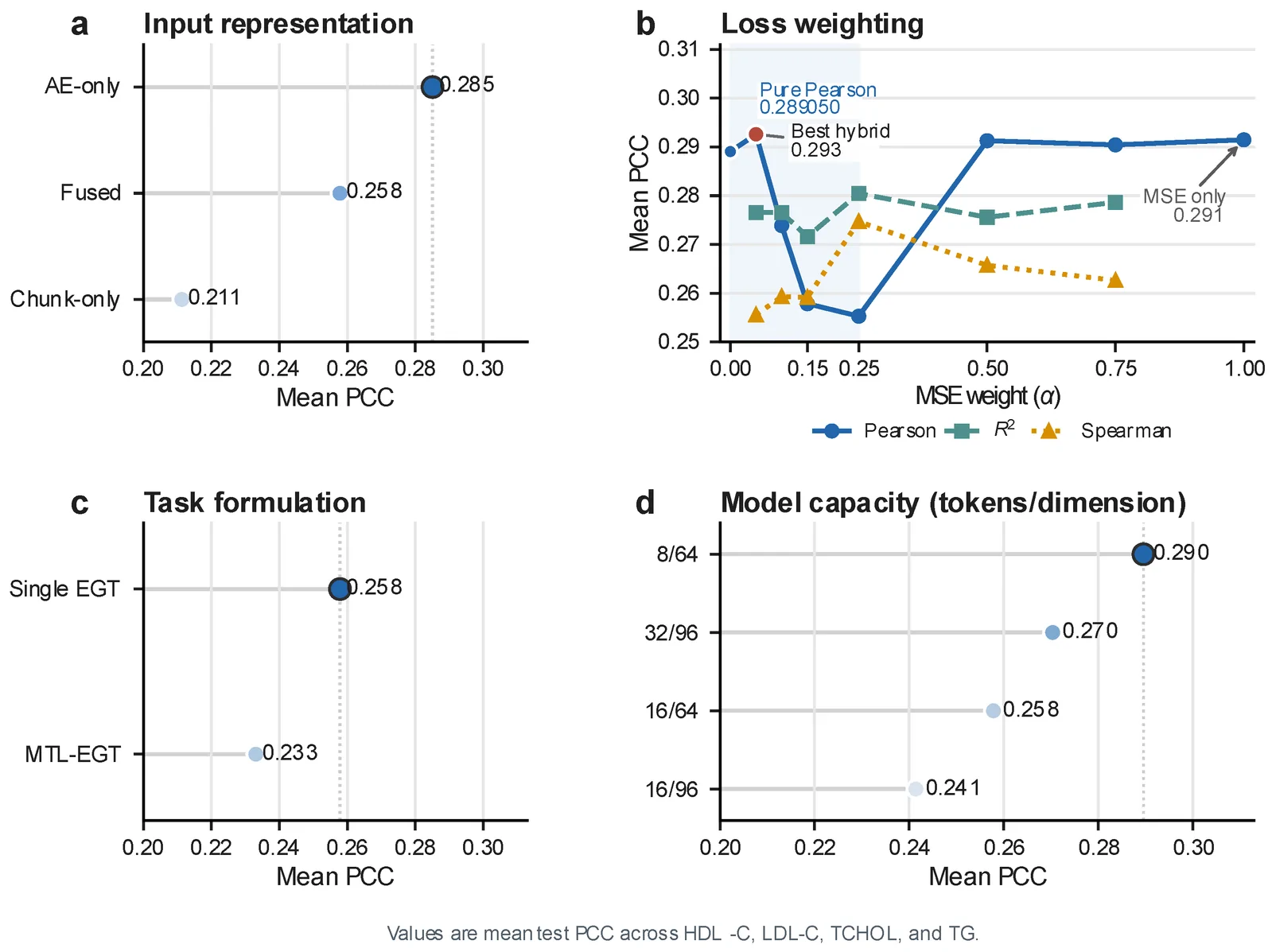
BloodLipid ablation results. Panels show the mean test-set PCC across HDL-C, LDL-C, TCHOL, and TG for input representations, MSE combined with Pearson, Spearman, or $R^{2}$ score terms, single- and multi-task training, and model capacity. The MSE weight is α and the score weight is 1−α; α=1 corresponds to MSE-only training. The vertical dotted lines in panels (**a**,**c**,**d**) mark the best mean PCC of the panel, and the shaded band in panel (**b**) marks the range of MSE weights detailed in Table 5. In panel (**b**) the solid line with circles denotes the Pearson score term, the dashed line with squares denotes $R^{2}$, and the dotted line with triangles denotes Spearman; the red point marks the selected setting.

The BloodLipid dataset was selected for ablation because, unlike PIC with anonymous production traits and PigHeaT with three traits measured under different production conditions, it provides four directly measured serum lipid traits within a single dataset. This setting enables input representation, task formulation, model capacity, and loss-function settings to be compared under the same forward-validation design, while allowing integrated-gradient and masking results for LDL-C, TCHOL, and TG to be interpreted for defined lipid phenotypes (Table 4; Figure 3 and Figure 5). AE-only, chunk-only, and fused inputs were compared using a common 16-token/64-dimensional EGT backbone, and the capacity comparison evaluated the 8/64 main configuration together with 16/64, 16/96, and 32/96 alternatives. AE-only, the global representation used in the main EGT, reached a mean PCC of 0.285, compared with 0.258 for the fused global–local representation and 0.211 for chunk-only input. Thus, the global AE-only representation performed better than the fused and chunk-only alternatives. These comparisons are reported as preliminary observations from this ablation family. In the task-formulation comparison using the same backbone, single-trait EGT reached 0.258, whereas MTL-EGT reached 0.233. The two representations also diverge on scale error: chunk-only input attains the lower mean MAE (0.203 against 0.206), whereas AE-only attains the higher PCC (0.285 against 0.211). The two metrics answer different questions and do not carry equal weight for this application. The models are used to rank and select candidate animals, so the selection decisions and the genetic gain they produce depend on the order in which animals are ranked, which is what PCC measures, whereas MAE is dominated by the absolute scale of the predictions. We therefore report both metrics and interpret the ablation primarily through PCC, which is also the metric on which the models are compared with the baselines.

Capacity differences were smaller than the representation differences. The 8-token/64-dimensional configuration gave the highest mean PCC (0.290), followed by 32/96 (0.270), 16/64 (0.258), and 16/96 (0.241). Increasing token count or embedding dimension did not consistently improve mean PCC.

The BloodLipid ablation experiments included Pearson correlation, a differentiable soft-rank Spearman correlation with temperature 0.1 [43], and $R^{2}=1-\mathrm{SSE}/\mathrm{SST}$ as alternative score terms. Pearson correlation measures linear agreement between observed and predicted phenotypes. Spearman correlation measures global rank agreement between observed and predicted phenotypes. $R^{2}$ measures goodness of fit. MSE controls absolute prediction error and prediction scale, whereas the three score terms quantify different aspects of agreement between observed and predicted phenotypes.

The loss-function comparison evaluated Pearson, Spearman, and $R^{2}$ score terms at different MSE weights (Table 5). The Pearson–MSE configuration with α=0.05 produced the highest mean PCC, 0.293, and the lowest mean RMSE and MAE, 0.253 and 0.199; the margin over MSE-only training is 0.002, which is smaller than the seed-to-seed variation of the replicated benchmark, so this ranking is reported as a preliminary observation. MSE-only training reached 0.291, Pearson-only training reached 0.289, and the Pearson–MSE configuration with α=0.15 reached 0.258. The strongest $R^{2}$ setting reached 0.280, whereas the strongest Spearman setting reached 0.275. The Pearson–MSE configuration with α=0.05 was selected as the best-performing setting in this comparison.

### 3.4. Genomic Regions Highlighted by Attribution Analysis

**Figure 6:**
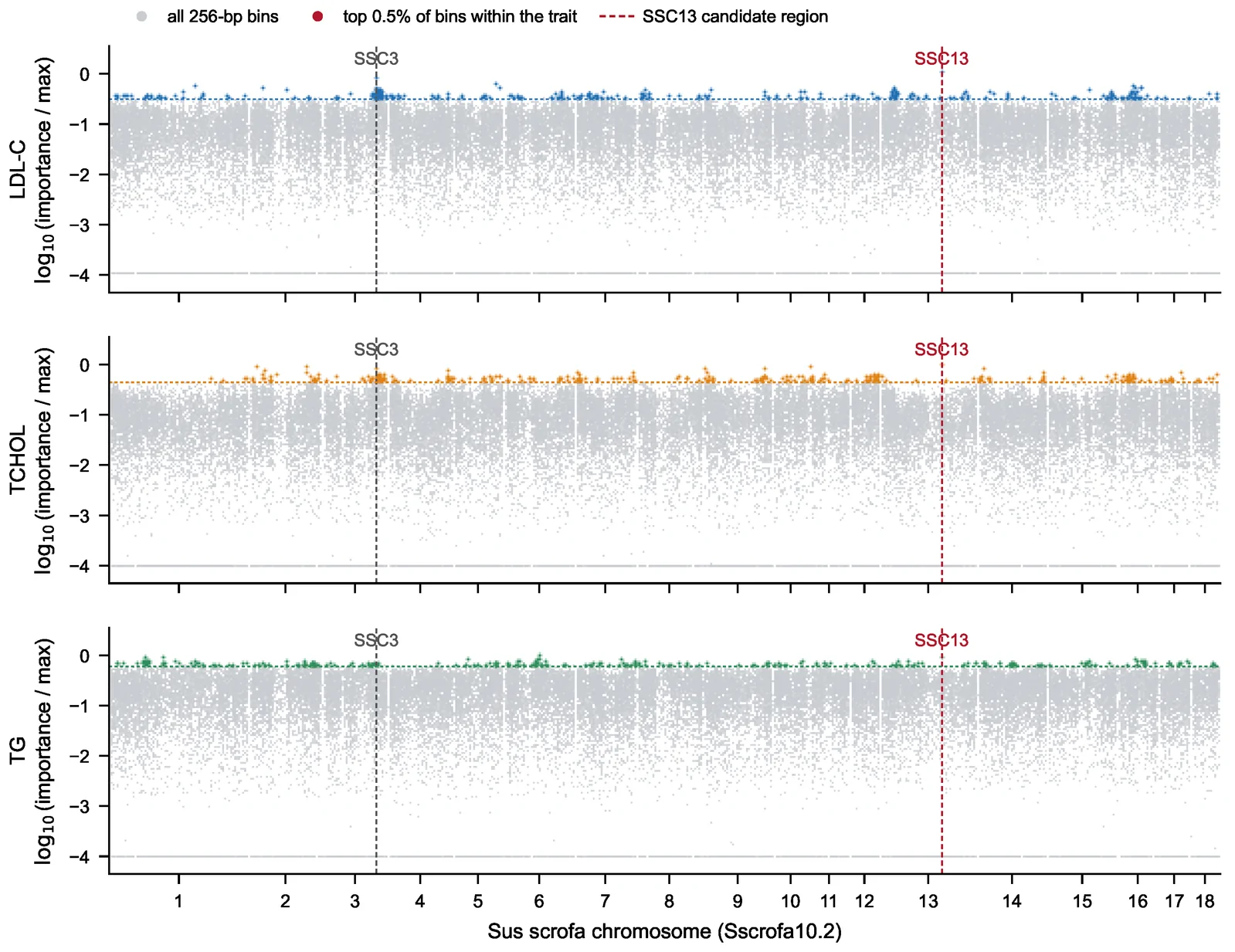
Genome-wide integrated-gradient importance for LDL-C, TCHOL and TG on the Sscrofa10.2 assembly (GCA_000003025.4). SNP-level absolute attributions were averaged across test animals and aggregated by coordinate into 256-bp bins. Each trait was normalized to its own maximum before log transformation. Unplaced markers, which carry no coordinate, and the non-chromosomal labels 23 and 24 are excluded, so the axis spans SSC1 to SSC18. The recurrent SSC13 candidate region near 140.5 Mb and the model’s SSC3 attribution near 121.75 Mb are marked.

Integrated gradients highlighted a recurrent region on *Sus scrofa* chromosome 13 (SSC13) near 140.5 Mb for LDL-C, TCHOL, and TG (Figure 6; Table 6). Marker MARC0013088 occurred among the representative high-importance markers for all three traits, whereas chromosome 5 for LDL-C, chromosome 2 for TCHOL, and chromosome 1 for TG provided trait-specific comparators.

On the Sscrofa10.2 assembly the marker maps to SSC13:140,497,749 and lies within an intron of the porcine *CFAP91* transcript (140,437,096 to 140,566,877); this intronic position is the genomic context reported for the marker throughout this study. In the 256-bp bin that holds it, [140,497,664,140,497,920), it is the only marker, and the nearest annotated lipid-metabolising gene is *PLA1A* (140,638,521 to 140,665,961), 141 kb away, with *COX17* and *POPDC2* between (Table 6). Across all 1259 animals and all 41 markers within 1 Mb, the marker’s maximum $r^{2}$ to any neighbour is 0.011 and the median is 0.003, so the attributed signal localises to a single variant. The variant is carried by 71 animals across the cohort.

The marker is not a proxy for population structure: its dosage correlates with the leading principal components at |r|≤0.109, and correcting the phenotypes for the first ten principal components leaves the attribution ranking of the marker unchanged. The model also recovers a published signal: its SSC3 attribution at about 121.75 Mb lies within about 3 Mb of the published SSC3 peak, where 106 of the 122 suggestive SNPs for these animals are located. Of the 43 curated records that overlap the SSC13 candidate region in the Animal QTLdb deposition, 12 (28%) concern circulating lipids or fatty-acid content—cholesterol level, LDL cholesterol, HDL cholesterol or the HDL/LDL ratio, triglyceride level, and monounsaturated fatty-acid content—while the remaining 31 describe backfat thickness, fat area or intramuscular fat, so the region is dominated by fat-deposition records with a lipid minority (Table S10), and 12 (28%) concern circulating lipids or fatty-acid content—cholesterol level, LDL cholesterol, HDL cholesterol or the HDL/LDL ratio, triglyceride level, and monounsaturated fatty-acid content—while the remaining 31 describe backfat thickness, fat area or intramuscular fat, so the region is dominated by fat-deposition records with a lipid minority (Table S10).

### 3.5. Masking of Ranked Attribution Bins

**Figure 7:**
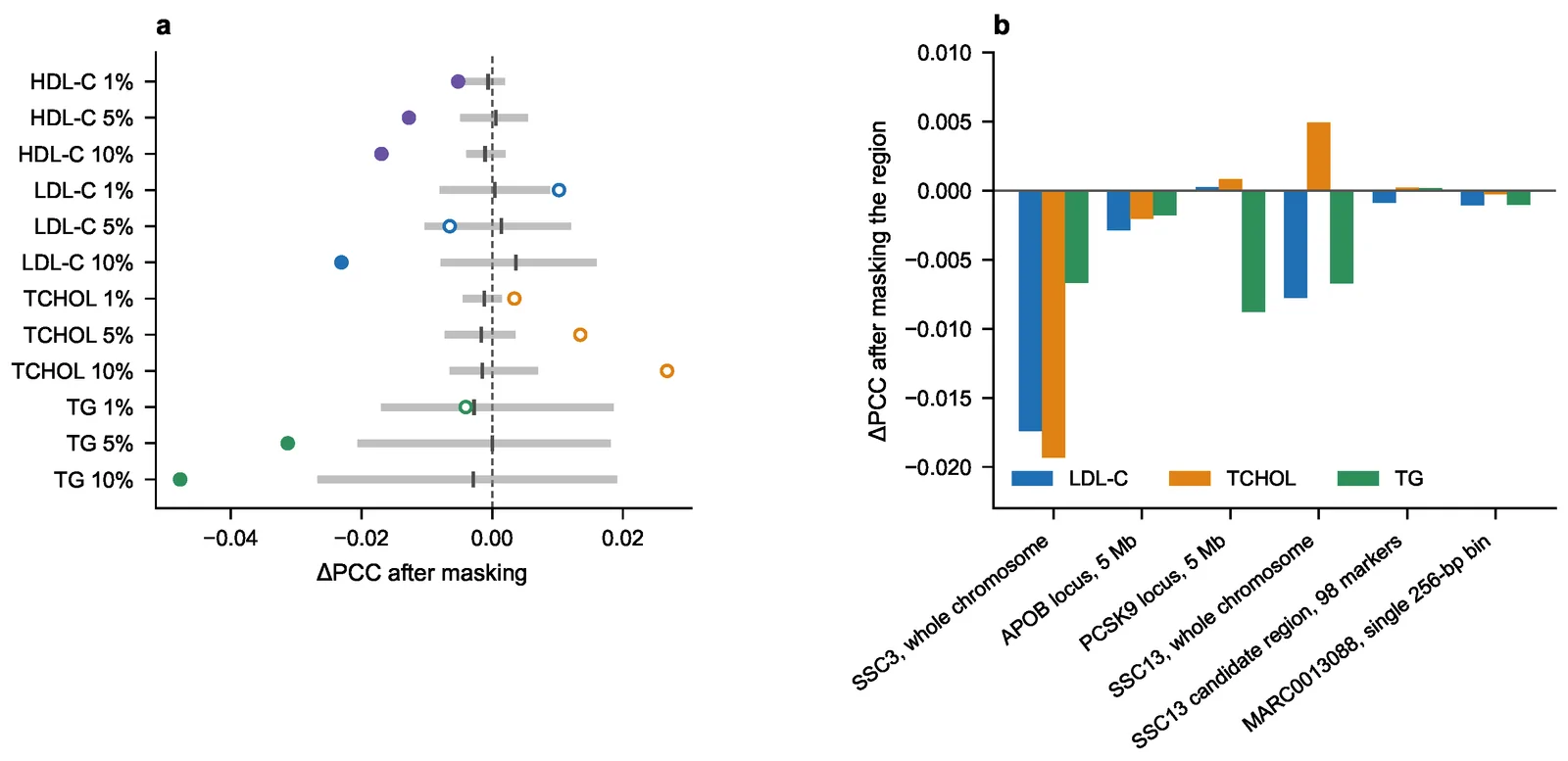
Masking analysis for BloodLipid attribution rankings. (**a**) Effect of masking the top-ranked bins on test PCC (markers) against a marker-count-matched random control (grey bars, 2.5 to 97.5 percentile interval over 20 independent draws); filled markers mark the comparisons in which the top-ranked bins fall below the control interval, which happens for HDL-C at all three fractions, for LDL-C at 10% and for TG at 5% and 10%, and not for TCHOL at any fraction. Purple, blue, orange and green markers denote HDL-C, LDL-C, TCHOL and TG, respectively. Filled circles mark the comparisons in which masking reduced test PCC below the control interval, and open circles mark the remaining comparisons. The short grey vertical marks are the control means, and the dashed vertical line marks zero change. (**b**) Locus-scale controls: masking the whole of SSC3, which carries the published *APOB* signal for these animals, and the SSC13 candidate region at chromosome, region and single-bin scale; the SSC13 controls move test PCC by at most 0.008.

Masking the top-ranked attribution bins reduced test-set PCC more than a marker-count-matched random control, but the reduction is confined to particular traits and masking fractions rather than being general (Figure 7a; Table S11). The reduction falls below the control interval in six of the twelve trait–fraction combinations: all three fractions for HDL-C (−0.005, −0.013 and −0.017), LDL-C at 10% (−0.023), and TG at 5% and 10% (−0.031 and −0.048). In the opposite direction the shift lies above the control interval for LDL-C at 1% (+0.010) and for TCHOL at all three fractions (+0.003, +0.014 and +0.027), whereas LDL-C at 5% and TG at 1% lie inside it. The locus-scale controls show that the assay detects a predictive region when one is masked, since masking SSC3 was the most damaging single region for LDL-C and TCHOL, whereas every SSC13 control is inert: masking the whole chromosome moves test PCC by at most 0.008 and the candidate region or the focal marker’s window by at most 0.0011 (Table S12).

We also recorded the computational cost of every model. EGT trains in 23.7±4.1 s per trait as a single-trait model and 29.1±5.1 s as MTL-EGT, requires 2.60 GB of GPU memory, and is the fastest model at inference (0.021 s per test split). Table 7 reports training time, inference time and peak GPU memory for all eight models.

## 4. Discussion

This study evaluated a compact EGT and six established prediction models across three pig datasets using forward-validation test sets, and compared MSE-only training with MSE combined with differentiable score terms. Across the 12 tasks, an EGT variant achieved a higher test-set PCC than the baseline in 10 of them, with the largest gain on LDL-C, and the magnitude of improvement varied substantially across traits. The variant and the baseline are both the best-performing choice of each cell, under one and the same rule, so the differences are read here as a per-trait comparison rather than as a uniform advantage. The trait-specific results support selecting prediction models separately for different breeding targets.

EGT was evaluated alongside CNN, residual, recurrent, and attention-based models that have been used for genomic prediction [12,14,15,16]. In the present study, continuous SNP genotype values from the PIC, PigHeaT, and BloodLipid datasets were compressed into autoencoder tokens, and the resulting EGT was evaluated with single-trait and multi-task prediction heads under dataset-specific forward-validation test sets. ChrFormer used chromosome encoding and ten-fold cross-validation for four growth traits [26]. In contrast, the present study evaluated EGT across three pig datasets and 12 prediction tasks using dataset-specific forward-validation test sets. This design broadens the assessment beyond growth traits and evaluates EGT under validation datasets that reflect prediction for later animals.

The multi-task and representation analyses showed that the simpler EGT design performed better than more complex alternatives. MTL-EGT improved several traits, whereas the BloodLipid ablation of single- and multi-task heads within the same EGT architecture favored single-trait training. The global AE-only representation outperformed both chunk-only and fused inputs, even though the latter added local marker information. The compact 8-token/64-dimensional configuration also achieved the highest mean PCC among the evaluated capacity settings. The BloodLipid ablation experiments favored AE-only input over chunk-only and fused inputs, single-trait training over multi-task training, and the 8-token/64-dimensional configuration over the other capacity settings.

The comparison of MSE and score terms showed that the Pearson–MSE configuration with α=0.05 achieved the best BloodLipid performance. The margin over MSE-only training is 0.002, smaller than the seed-to-seed variation of the replicated benchmark in Table 2, so the selection of α=0.05 is reported as a preliminary observation; the same applies to the preference for the AE-only representation. This setting increased mean PCC from 0.291 for MSE-only training to 0.293 and produced the lowest mean RMSE and MAE. MSE controls numerical prediction error and prediction scale, whereas Pearson, Spearman, and $R^{2}$ score terms quantify linear agreement, rank agreement, and goodness of fit, respectively.

Attribution analysis highlighted an SSC13 region near 140.5 Mb as a shared high-importance region for LDL-C, TCHOL, and TG. MARC0013088 recurred among the high-importance markers for all three traits, identifying this region as a priority for annotation and targeted validation [44]. Masking the top-ranked bins reduced test PCC more than a marker-count-matched random control for HDL-C at all three fractions and for TG and LDL-C at the 5–10% fractions, whereas masking TCHOL bins raised test PCC at all three fractions, above the control interval, and whole-chromosome controls confirmed that the assay detects a predictive region when one is masked. The effect is therefore trait- and fraction-specific rather than general, and the conservative reading is that the attribution ranking identifies features the fitted models use in some traits but that the ranking alone does not establish predictive dependence. For SSC13 the same assay is inert: masking the whole chromosome, the candidate region or the marker’s own window moves test PCC by at most 0.008, so the region carries attribution but no measurable predictive dependence and is reported as a candidate flagged by attribution rather than as a validated locus. Positional annotation placed the marker within an intron of the porcine *CFAP91* transcript, with *PLA1A* as the nearest annotated lipid-metabolising gene, 141 kb away.

The public datasets are modest in size and cover only the breeds, farms, genotyping platforms, and management conditions represented in this study. Interpretability analyses were conducted for LDL-C, TCHOL, and TG in the BloodLipid dataset.

The correlation between predicted and observed performance is the accuracy of selection scaled by the root heritability, and expected genetic gain is proportional to that accuracy, so a relative difference in PCC corresponds to approximately the same relative difference in expected selection response. On TG, where the difference is +0.041 against accuracies of 0.595 and 0.554, this corresponds to roughly a 7% relative difference in ranking accuracy, and the largest differences in this study fall on the lipid traits.

Deployment in a commercial programme depends on more than ranking accuracy. EGT trains within seconds per trait, requires 2.60 GB of GPU memory and is the fastest model at inference, which makes repeated retraining practical. Single-step GBLUP additionally integrates ungenotyped relatives through pedigree relationships, so the two approaches are complementary: EGT is competitive on a subset of traits and extends the available toolkit, with the advantage reported trait by trait and largest where predictability is moderate to high.

The compression behind the slopes of Table 3 matters for breeding use. A correlation objective is invariant to a positive rescaling of the predictions, so it does not penalise compressed values, and MSE does not undo shrinkage towards the training mean on a small reference population. Within-cohort ranking is unaffected, because a monotone rescaling does not change the order of a cohort’s candidates, but any other use does: Selection-index weights and any comparison of a young genomic candidate against a proven animal with a measured phenotype require predictions on the observed scale with a defensible spread, so a post-hoc calibration step, such as rescaling with the slope of true on predicted value estimated on the validation dataset, would be needed before these outputs are used as selection criteria. Slopes are reported for every cell so that such a step can be applied and audited, and we do not claim that the raw outputs are ready for a selection index. A correlation objective is invariant to a positive rescaling of the predictions, so it does not penalise compressed values, and MSE does not undo shrinkage towards the training mean on a small reference population. Within-cohort ranking is unaffected, because a monotone rescaling does not change the order of a cohort’s candidates, but any other use does: Selection-index weights and any comparison of a young genomic candidate against a proven animal with a measured phenotype require predictions on the observed scale with a defensible spread, so a post-hoc calibration step, such as rescaling with the slope of true on predicted value estimated on the validation dataset, would be needed before these outputs are used as selection criteria. Slopes are reported for every cell so that such a step can be applied and audited, and we do not claim that the raw outputs are ready for a selection index. One cell of the benchmark favours a baseline significantly, PIC t2. The trait is one of the anonymously labelled PIC variables reconstructed from a released file, and the random forest that wins it can split on whichever interaction structure dominates that variable, whereas the compressed token representation averages over the genome.

## 5. Conclusions

This study addresses the need to assess pig genomic prediction models using validation datasets that reflect prediction for later animals. We constructed a forward-validation benchmark across 12 prediction tasks from the PIC, PigHeaT, and BloodLipid datasets. We then developed EGT, which compresses genome-wide SNP genotypes into autoencoder-derived tokens before self-attention. In comparative experiments, an EGT variant achieved a higher test-set PCC than the baseline in 10 of the 12 tasks. BloodLipid ablations showed that performance differed among input representations, task formulations, model capacities, and loss-function settings. The Pearson–MSE objective achieved the highest mean PCC and the lowest mean RMSE and MAE in the BloodLipid loss-function comparison. Integrated gradients highlighted a recurrent SSC13 candidate region across LDL-C, TCHOL, and TG and identified a lipid-related candidate region for follow-up, although the masking controls detected no measurable effect of this region on test-set predictions. These findings provide a forward-validation benchmark for selecting genomic prediction models and prioritizing genomic regions in pig breeding. Taken together, the results position EGT as a trait-dependent complement to established evaluation rather than a replacement for it; the compressed prediction range of Table 3 means that post-hoc calibration is required before these outputs are used outside within-cohort ranking.

## Figures and Tables

**Table 1:** Datasets, evaluated traits and forward-validation partitions. PIC trait labels (t1–t5) follow the anonymous identifiers in the public dataset release.

Dataset	Traits	Animals	SNPs	Train/Valid/Test	Ordering for Forward Validation
PigHeaT	BFT19, RT1923, RFI	1148	50,735	804/122/222	birth year, 2013 train and validation to 2014 test
PIC	t1, t2, t3, t4, t5	3534	52,843	2473/354/707	pedigree-generation depth
BloodLipid	HDL-C, LDL-C, TCHOL, TG	1259	61,565	881/125/253	experimental batch within each population

*Note:* Test-set sizes differ between traits according to record availability and are therefore smaller than the nominal partition sizes: 222 for PigHeaT, 250–251 for BloodLipid and 286–688 for PIC. The exact number of tested animals is given for every trait in Table S1.

**Table 2:** Predictive performance on the forward-validation test sets. Values are the mean ± standard deviation over five replicate seeds; the confidence interval is a paired bootstrap (B = 10,000) resampling both test animals and replicate seeds.

Dataset	Trait	EGT Variant	EGT PCC Mean ± SD	Baseline	Baseline PCC Mean ± SD	ΔPCC [95% CI]
PigHeaT	BFT19	Single-EGT	0.549±0.030	GBLUP	0.514±0.000	**+0.035** [+0.002, +0.076]
PigHeaT	RT1923	Single-EGT	0.149±0.014	RF	0.142±0.028	+0.007 [−0.010, +0.024]
PigHeaT	RFI	Single-EGT	0.179±0.009	GBLUP	0.173±0.000	**+0.006** [+0.0003, +0.022]
BloodLipid	HDL-C	MTL-EGT	0.085±0.007	SVR	0.060±0.000	**+0.025** [+0.016, +0.034]
BloodLipid	LDL-C	MTL-EGT	0.181±0.033	XGBoost	0.124±0.014	**+0.057** [+0.013, +0.102]
BloodLipid	TCHOL	Single-EGT	0.309±0.020	SVR	0.294±0.000	**+0.015** [+0.006, +0.056]
BloodLipid	TG	Single-EGT	0.595±0.033	GBLUP	0.554±0.000	**+0.041** [+0.00003, +0.082]
PIC	t1	MTL-EGT	0.046±0.032	CNN	0.061±0.048	−0.015 [−0.087, +0.057]
PIC	t2	MTL-EGT	0.091±0.017	RF	0.120±0.016	**−0.029** [−0.058, −0.00002]
PIC	t3	MTL-EGT	0.075±0.011	RF	0.043±0.020	**+0.032** [+0.004, +0.060]
PIC	t4	MTL-EGT	0.345±0.018	GBLUP	0.308±0.000	**+0.037** [+0.015, +0.059]
PIC	t5	MTL-EGT	0.183±0.032	GBLUP	0.149±0.000	**+0.034** [+0.002, +0.082]

*Note:* The EGT column reports the better of the single-trait and multi-task variants and the baseline column the strongest of the six conventional models, so that the two are compared at their best under one and the same rule; both EGT variants were trained for every dataset and trait, and the per-model values of Table S1 allow the comparison to be repeated against any of the six baselines. ΔPCC is the EGT variant minus the baseline. Bold ΔPCC marks the cells whose interval excludes zero: 10 of 12, nine favouring EGT and one favouring the baseline (PIC t2); the two remaining cells are PigHeaT RT1923 and PIC t1. An SD of 0.000 denotes a deterministic estimator, whose predictions are identical across replicate seeds. Per-model values for all eight models and the per-trait test-set sizes are given in the Supplementary Material.

**Table 3:** Prediction bias and regression slope for the EGT variant reported in Table 2 and for the baseline of each cell. Both statistics are computed on the original phenotypic scale, after the standardized predictions are returned to that scale.

Dataset	Trait	Vs.	Slope EGT	Slope Baseline	|Slope −1| EGT	|Slope −1| Baseline	|Bias|/SD EGT	|Bias|/SD Baseline
PigHeaT	BFT19	GBLUP	0.751	0.850	0.249	0.150	**0.025**	0.032
PigHeaT	RT1923	RF	0.340	1.063	0.660	0.063	**0.017**	0.027
PigHeaT	RFI	GBLUP	0.340	5.001	0.660	4.001	0.034	**0.004**
BloodLipid	HDL-C	SVR	0.099	0.066	0.901	0.934	0.308	**0.093**
BloodLipid	LDL-C	XGBoost	0.164	0.476	0.836	0.524	**0.014**	0.035
BloodLipid	TCHOL	SVR	0.500	0.403	0.500	0.597	**0.083**	0.168
BloodLipid	TG	GBLUP	1.259	3.306	0.259	2.306	**0.060**	0.110
PIC	t1	CNN	0.092	2.901	0.908	1.901	0.537	**0.026**
PIC	t2	RF	0.198	1.443	0.802	0.443	**0.094**	0.552
PIC	t3	RF	0.153	0.466	0.847	0.534	**0.026**	0.277
PIC	t4	GBLUP	0.386	1.084	0.614	0.084	0.294	**0.107**
PIC	t5	GBLUP	0.296	1.182	0.704	0.182	**0.166**	0.380

*Note:* Bias is the mean of (predicted − true), divided by each trait’s phenotypic standard deviation estimated on the training dataset, because the twelve traits span standard deviations from 0.23 to 336.5 and raw bias values are therefore not comparable across cells. Slope is the regression of true phenotype on predicted value, computed after the predictions are returned to the original phenotypic scale ($\hat{y}={\hat{y}}^{\;\mathrm{std}}\;{\mathrm{SD}}_{\mathrm{train}}+{\mathrm{mean}}_{\mathrm{train}}$); the slopes therefore describe variance compression of the predictions, not an arbitrary scaling of standardized units. Bold marks the smaller absolute bias of the pair. EGT’s bias is smaller in eight of the twelve cells, with a mean absolute bias of 0.138 SD against 0.151 SD. Values are means over the five replicate seeds for the EGT side and over the runs available for the baseline side, which is a single run for the deterministic estimators and for the PigHeaT baseline block.

**Table 4:** BloodLipid ablation results for input representation, task formulation and model capacity.

*Shared Reference Configuration:* Fused Representation, Single-Trait EGT, 16 Tokens/64 Dimensions, Seed 42				
Section	Setting	Mean PCC	Mean RMSE	Mean MAE
Reference	Shared reference configuration	**0.258**	0.277	0.219
Representation design	AE-only (global)	**0.285**	**0.260**	0.206
	Chunk-only (local)	0.211	0.262	**0.203**
Task formulation	MTL-EGT	0.233	**0.260**	**0.205**
Capacity sensitivity	8/64 (main analysis)	**0.290**	**0.254**	**0.199**
	16/96	0.241	0.269	0.209
	32/96	0.270	0.257	0.200

*Note:* Capacity settings are reported as token count/embedding dimension; values are means over the four BloodLipid traits. Bold marks the best value per column within each family.

**Table 5:** BloodLipid comparison of MSE and score-term settings. The selected setting and its immediate neighbourhood are shown; the complete weight grid is given in the Supplementary Material.

Score Term	MSE Weight (α)	Score Weight (1−α)	Mean PCC	Mean RMSE	Mean MAE
Pearson (score only)	0.00	1.00	0.289	0.259	0.213
Pearson	0.05	0.95	**0.293**	**0.253**	**0.199**
Pearson	0.15	0.85	0.258	0.277	0.219
MSE only	1.00	0.00	0.291	0.267	0.208
$R^{2}$ (strongest setting)	0.25	0.75	0.280	0.268	0.208
Spearman (strongest setting)	0.25	0.75	0.275	0.272	0.214

*Note:* Values are means over the four BloodLipid traits from single runs without replicates, so they carry no uncertainty estimate; bold marks the setting with the highest mean PCC, α=0.05 with a Pearson score term, which is used for the reported models. The difference from MSE-only training (0.293 against 0.291) is smaller than the seed-to-seed variation of the replicated benchmark in Table 2, so the ranking of neighbouring settings is a preliminary empirical observation rather than a demonstrated improvement.

**Table 6:** Representative high-importance markers and porcine positional annotations from the BloodLipid integrated-gradients analysis. Coordinates are given on the Sscrofa10.2 assembly (GCA_000003025.4).

Trait	Bin (chr:bp)	Marker	IG Score	Shared	Gene	Annotation
LDL-C	13:140497664–140497920	MARC0013088	0.137	Yes	*CFAP91* (intronic)	Intronic *CFAP91*; nearest annotated lipid-metabolism gene *PLA1A*, 141 kb away
LDL-C	5:95090432–95090688	DRGA0006183	0.082	No	—	Trait-specific LDL-C signal without recurrent cross-trait support
TCHOL	13:140497664–140497920	MARC0013088	0.639	Yes	*CFAP91* (intronic)	Shared SSC13 candidate region, strongest cross-trait signal of the three traits
TCHOL	2:129405696–129405952	ALGA0015662	0.550	No	—	Trait-specific TCHOL signal outside the shared SSC13 candidate region
TG	1:78526976–78527232	INRA0002835	0.024	No	—	Trait-specific TG signal outside the shared SSC13 candidate region
TG	13:140497664–140497920	MARC0013088	0.024	Yes	*CFAP91* (intronic)	Shared SSC13 candidate region with consistent recurrence across lipid traits

*Note:* Bins are half-open 256-bp genomic intervals, [start,end), aggregated by genomic coordinate; with 61,565 array markers at an average spacing of roughly 40 kb, such a bin contains on average about one marker, and the bin holding the top-ranked marker contains exactly one. The integrated-gradient (IG) score is the mean absolute SNP-level attribution across test animals, and “Shared” denotes recurrence of the named marker across the three lipid traits. The region is reported as a candidate flagged by model attribution rather than as a validated QTL.

**Table 7:** Computational cost of the eight models over the complete replicate sample.

Model	Training Time (s)	Inference Time (s, Test Split)	Peak GPU Memory (GB)
RNN	5.9 ± 2.2	0.072	1.58
GBLUP	8.3 ± 5.3	0.240	—
Random Forest	9.8 ± 4.9	0.214	—
EGT (single-trait)	23.7 ± 4.1	**0.021**	2.60
XGBoost	27.0 ± 16.3	0.356	—
EGT (multi-task)	29.1 ± 5.1	**0.022**	2.60
CNN	38.1 ± 21.2	0.144	6.73
SVR	54.2 ± 47.7	0.226	—

*Note:* All models were timed on the same machine. GBLUP, SVR and Random Forest ran on the CPU (AMD Ryzen Threadripper PRO 7975WX, 32 cores); EGT, CNN, RNN and XGBoost ran on the GPU (NVIDIA RTX A6000). Training times are the mean ± standard deviation over the replicate runs; inference is the mean time to score one test split. A dash denotes a model that does not use the GPU. Bold inference times mark the two fastest models.

## Data Availability

PIC was derived from the benchmark dataset described by Cleveland et al. [5]. PigHeaT was derived from the public PigHeaT dataset, available at https://doi.org/10.57745/TLKLRJ [7]. BloodLipid was derived from the Dryad release associated with Yang et al., available at https://doi.org/10.5061/dryad.4gh70 [8]. Processed datasets prepared as model inputs for this study are available as compressed archives at https://github.com/xwx1999/EGT (accessed on 13 October 2026). The code used for preprocessing, baseline benchmarking, EGT training, and explainability is publicly available at the same repository, and the archived release is permanently available at Zenodo: https://doi.org/10.5281/zenodo.22977074.

## Supplementary Materials

The following supporting information can be downloaded at https://www.mdpi.com/article/10.3390/ani16203284/s1, Table S1: Test-set PCC for every dataset, trait and model, with the exact test-set sample size of each trait; Table S2: Prediction bias and regression slope for every dataset, trait and model; Table S3: Marker quality control applied to each dataset and the number of markers that a minor-allele-frequency and call-rate mask would remove; Table S4: Phenotype distributions of the retained traits; Table S5: Local linkage disequilibrium around MARC0013088; Table S6: Allele frequency of MARC0013088 by population; Table S7: Effect of correcting the phenotypes for population structure on test-set PCC; Table S8: Attribution of MARC0013088 before and after correcting for population structure; Table S9: Comparison with the published genome-based GWAS results for these animals; Table S10: Animal QTLdb records overlapping the SSC13 candidate region; Table S11: Repeated masking of the top-attributed bins against a marker-count-matched random control; Table S12: Locus-scale controls for the masking analysis; Table S13: Complete BloodLipid loss-weight grid; Table S14: Optimisation settings for every model; Table S15: The reported comparison under two selection rules, one that takes each side at its best on the test dataset and one that takes each side at its best on the validation dataset; Table S16: Validation-set PCC of every model for the twelve reported traits.
