# Supplementary material for: An Enhanced Genomic Transformer for Multi-Dataset Forward-Validation Benchmarking in Pig Genomic Prediction
Source: Animals (Basel). 2026 Oct 18;16(20):3284. doi: 10.3390/ani16203284 (PMC13700791; doi:10.3390/ani16203284)
Supplement: Supplementary file 1 [file animals-16-03284-s001.zip › animals-4559967-supplementary.pdf]

Supplementary tables

Table S1: Test-set PCC for every dataset, trait and model, and the exact test-set sample size of each trait.

Dataset	Trait	Model	Test PCC	Test animals
PigHeaT	BFT19	GBLUP	0.514 ± 0.000	222
PigHeaT	BFT19	SVR	0.421 ± 0.000	222
PigHeaT	BFT19	RF	0.464 ± 0.024	222
PigHeaT	BFT19	XGBoost	0.502 ± 0.032	222
PigHeaT	BFT19	CNN	0.160 ± 0.016	222
PigHeaT	BFT19	RNN	0.225 ± 0.049	222
PigHeaT	BFT19	Single-EGT	0.549 ± 0.030	222
PigHeaT	BFT19	MTL-EGT	0.526 ± 0.007	222
PigHeaT	RT1923	GBLUP	0.114 ± 0.000	221
PigHeaT	RT1923	SVR	0.091 ± 0.000	221
PigHeaT	RT1923	RF	0.142 ± 0.028	221
PigHeaT	RT1923	XGBoost	0.046 ± 0.009	221
PigHeaT	RT1923	CNN	0.004 ± 0.003	221
PigHeaT	RT1923	RNN	0.012 ± 0.007	221
PigHeaT	RT1923	Single-EGT	0.149 ± 0.014	221
PigHeaT	RT1923	MTL-EGT	0.125 ± 0.043	221
PigHeaT	RFI	GBLUP	0.173 ± 0.000	220
PigHeaT	RFI	SVR	0.091 ± 0.000	220
PigHeaT	RFI	RF	0.161 ± 0.017	220
PigHeaT	RFI	XGBoost	0.145 ± 0.032	220
PigHeaT	RFI	CNN	0.015 ± 0.007	220
PigHeaT	RFI	RNN	0.047 ± 0.013	220
PigHeaT	RFI	Single-EGT	0.179 ± 0.009	220
PigHeaT	RFI	MTL-EGT	0.141 ± 0.050	220
BloodLipid	HDL-C	GBLUP	0.054 ± 0.000	250
BloodLipid	HDL-C	SVR	0.060 ± 0.000	250
BloodLipid	HDL-C	RF	0.043 ± 0.004	250
BloodLipid	HDL-C	XGBoost	0.014 ± 0.012	250
BloodLipid	HDL-C	CNN	0.040 ± 0.005	250
BloodLipid	HDL-C	RNN	0.039 ± 0.031	250
BloodLipid	HDL-C	Single-EGT	0.045 ± 0.055	250
BloodLipid	HDL-C	MTL-EGT	0.085 ± 0.007	250
BloodLipid	LDL-C	GBLUP	0.102 ± 0.000	251
BloodLipid	LDL-C	SVR	0.108 ± 0.000	251
BloodLipid	LDL-C	RF	0.112 ± 0.010	251
BloodLipid	LDL-C	XGBoost	0.124 ± 0.014	251
BloodLipid	LDL-C	CNN	0.035 ± 0.039	251
BloodLipid	LDL-C	RNN	0.072 ± 0.027	251
BloodLipid	LDL-C	Single-EGT	0.125 ± 0.020	251
BloodLipid	LDL-C	MTL-EGT	0.181 ± 0.033	251
BloodLipid	TCHOL	GBLUP	0.289 ± 0.000	251
BloodLipid	TCHOL	SVR	0.294 ± 0.000	251
BloodLipid	TCHOL	RF	0.208 ± 0.012	251
BloodLipid	TCHOL	XGBoost	0.229 ± 0.016	251
BloodLipid	TCHOL	CNN	0.109 ± 0.043	251
BloodLipid	TCHOL	RNN	0.010 ± 0.048	251
BloodLipid	TCHOL	Single-EGT	0.309 ± 0.020	251
BloodLipid	TCHOL	MTL-EGT	0.294 ± 0.043	251
BloodLipid	TG	GBLUP	0.554 ± 0.000	251
BloodLipid	TG	SVR	0.436 ± 0.000	251
BloodLipid	TG	RF	0.514 ± 0.001	251
BloodLipid	TG	XGBoost	0.513 ± 0.015	251
BloodLipid	TG	CNN	0.502 ± 0.001	251
BloodLipid	TG	RNN	0.545 ± 0.030	251
BloodLipid	TG	Single-EGT	0.595 ± 0.033	251
BloodLipid	TG	MTL-EGT	0.526 ± 0.034	251
PIC	t1	GBLUP	0.013 ± 0.000	286
PIC	t1	SVR	0.018 ± 0.000	286
PIC	t1	RF	0.029 ± 0.016	286
PIC	t1	XGBoost	0.036 ± 0.031	286
PIC	t1	CNN	0.061 ± 0.048	286
PIC	t1	RNN	0.029 ± 0.107	286
PIC	t1	Single-EGT	0.044 ± 0.024	286
PIC	t1	MTL-EGT	0.046 ± 0.032	286
PIC	t2	GBLUP	0.052 ± 0.000	352
PIC	t2	SVR	0.058 ± 0.000	352
PIC	t2	RF	0.120 ± 0.016	352

Dataset	Trait	Model	Test PCC	Test animals
PIC	t2	XGBoost	0.062 ± 0.025	352
PIC	t2	CNN	0.106 ± 0.012	352
PIC	t2	RNN	0.064 ± 0.023	352
PIC	t2	Single-EGT	0.079 ± 0.020	352
PIC	t2	MTL-EGT	0.091 ± 0.017	352
PIC	t3	GBLUP	0.013 ± 0.000	688
PIC	t3	SVR	0.042 ± 0.000	688
PIC	t3	RF	0.043 ± 0.020	688
PIC	t3	XGBoost	0.026 ± 0.023	688
PIC	t3	CNN	0.026 ± 0.024	688
PIC	t3	RNN	0.010 ± 0.032	688
PIC	t3	Single-EGT	0.056 ± 0.025	688
PIC	t3	MTL-EGT	0.075 ± 0.011	688
PIC	t4	GBLUP	0.308 ± 0.000	688
PIC	t4	SVR	0.271 ± 0.000	688
PIC	t4	RF	0.302 ± 0.010	688
PIC	t4	XGBoost	0.284 ± 0.017	688
PIC	t4	CNN	0.157 ± 0.002	688
PIC	t4	RNN	0.216 ± 0.028	688
PIC	t4	Single-EGT	0.342 ± 0.007	688
PIC	t4	MTL-EGT	0.345 ± 0.018	688
PIC	t5	GBLUP	0.149 ± 0.000	688
PIC	t5	SVR	0.137 ± 0.000	688
PIC	t5	RF	0.143 ± 0.022	688
PIC	t5	XGBoost	0.128 ± 0.032	688
PIC	t5	CNN	0.011 ± 0.018	688
PIC	t5	RNN	0.094 ± 0.026	688
PIC	t5	Single-EGT	0.173 ± 0.008	688
PIC	t5	MTL-EGT	0.183 ± 0.032	688

Note: Values are test-set PCC for every dataset, trait and model, given as the mean ± standard deviation over the five replicate seeds (11, 17, 23, 29, 53). An SD of 0.000 marks a deterministic estimator, whose predictions are identical across replicate seeds. The Test animals column gives the exact number of animals with a record in the test dataset for that trait, which is why test sizes differ between traits of the same dataset. An SD of 0.000 marks a deterministic estimator, whose predictions are identical across replicate seeds.

Table S2: Prediction bias and regression slope for every dataset, trait and model.

Dataset	Trait	Model	Slope	$\vert$slope$-$1$\vert$	$\vert$bias$\vert$/SD
PigHeaT	BFT19	GBLUP	0.850	0.150	0.033
PigHeaT	BFT19	SVR	0.489	0.511	0.015
PigHeaT	BFT19	RF	1.715	0.715	0.044
PigHeaT	BFT19	XGBoost	1.112	0.112	0.001
PigHeaT	BFT19	CNN	2.833	1.833	0.049
PigHeaT	BFT19	RNN	0.656	0.344	0.088
PigHeaT	BFT19	Single-EGT	0.801	0.199	0.026
PigHeaT	BFT19	MTL-EGT	1.051	0.051	0.066
PigHeaT	RT1923	GBLUP	0.874	0.126	0.029
PigHeaT	RT1923	SVR	0.135	0.865	0.105
PigHeaT	RT1923	RF	1.063	0.063	0.029
PigHeaT	RT1923	XGBoost	0.150	0.850	0.064
PigHeaT	RT1923	CNN	0.065	0.935	0.208
PigHeaT	RT1923	RNN	0.053	0.947	0.108
PigHeaT	RT1923	Single-EGT	0.548	0.452	0.018
PigHeaT	RT1923	MTL-EGT	0.327	0.673	0.037
PigHeaT	RFI	GBLUP	5.001	4.001	0.004
PigHeaT	RFI	SVR	0.094	0.906	0.029
PigHeaT	RFI	RF	0.920	0.080	0.047
PigHeaT	RFI	XGBoost	0.384	0.616	0.015
PigHeaT	RFI	CNN	0.810	0.190	0.036
PigHeaT	RFI	RNN	0.223	0.777	0.014
PigHeaT	RFI	Single-EGT	0.381	0.619	0.035
PigHeaT	RFI	MTL-EGT	0.289	0.711	0.067
BloodLipid	HDL-C	GBLUP	0.060	0.940	0.403
BloodLipid	HDL-C	SVR	0.066	0.934	0.119
BloodLipid	HDL-C	RF	0.068	0.932	0.392
BloodLipid	HDL-C	XGBoost	0.020	0.980	0.396
BloodLipid	HDL-C	CNN	0.199	0.801	0.471
BloodLipid	HDL-C	RNN	0.045	0.955	0.347
BloodLipid	HDL-C	Single-EGT	0.055	0.945	0.319

Table S2: Prediction bias and regression slope for every dataset, trait and model.

Dataset	Trait	Model	Slope	|slope−1|	|bias|/SD
BloodLipid	HDL-C	MTL-EGT	0.103	0.897	0.395
BloodLipid	LDL-C	GBLUP	0.433	0.567	0.064
BloodLipid	LDL-C	SVR	0.238	0.762	0.359
BloodLipid	LDL-C	RF	0.490	0.510	0.055
BloodLipid	LDL-C	XGBoost	0.511	0.489	0.044
BloodLipid	LDL-C	CNN	0.221	0.779	0.629
BloodLipid	LDL-C	RNN	0.122	0.878	0.039
BloodLipid	LDL-C	Single-EGT	0.164	0.836	0.014
BloodLipid	LDL-C	MTL-EGT	0.251	0.749	0.013
BloodLipid	TCHOL	GBLUP	0.411	0.589	0.129
BloodLipid	TCHOL	SVR	0.403	0.597	0.192
BloodLipid	TCHOL	RF	0.793	0.207	0.041
BloodLipid	TCHOL	XGBoost	0.548	0.452	0.036
BloodLipid	TCHOL	CNN	2.935	1.935	0.069
BloodLipid	TCHOL	RNN	0.054	0.946	0.025
BloodLipid	TCHOL	Single-EGT	0.576	0.424	0.095
BloodLipid	TCHOL	MTL-EGT	0.409	0.591	0.109
BloodLipid	TG	GBLUP	3.306	2.306	0.095
BloodLipid	TG	SVR	0.749	0.251	0.462
BloodLipid	TG	RF	1.714	0.714	0.157
BloodLipid	TG	XGBoost	1.391	0.391	0.128
BloodLipid	TG	CNN	10.834	9.834	0.032
BloodLipid	TG	RNN	1.289	0.289	0.067
BloodLipid	TG	Single-EGT	1.339	0.339	0.052
BloodLipid	TG	MTL-EGT	0.905	0.095	0.026
PIC	t1	GBLUP	0.027	0.973	0.544
PIC	t1	SVR	0.018	0.982	0.453
PIC	t1	RF	0.397	0.603	0.141
PIC	t1	XGBoost	0.166	0.834	0.270
PIC	t1	CNN	2.986	1.986	0.021
PIC	t1	RNN	0.009	0.991	0.375
PIC	t1	Single-EGT	0.092	0.908	0.537
PIC	t1	MTL-EGT	0.156	0.844	0.459
PIC	t2	GBLUP	0.715	0.285	0.298
PIC	t2	SVR	0.052	0.948	0.355
PIC	t2	RF	1.645	0.645	0.644
PIC	t2	XGBoost	0.393	0.607	0.389
PIC	t2	CNN	0.785	0.215	0.122
PIC	t2	RNN	0.148	0.852	0.362
PIC	t2	Single-EGT	0.198	0.802	0.094
PIC	t2	MTL-EGT	0.223	0.777	0.041
PIC	t3	GBLUP	0.397	0.603	0.325
PIC	t3	SVR	0.032	0.968	0.219
PIC	t3	RF	0.681	0.319	0.358
PIC	t3	XGBoost	0.091	0.909	0.200
PIC	t3	CNN	0.477	0.523	0.485
PIC	t3	RNN	-0.005	1.005	0.077
PIC	t3	Single-EGT	0.170	0.830	0.112
PIC	t3	MTL-EGT	0.199	0.801	0.034
PIC	t4	GBLUP	1.084	0.084	0.111
PIC	t4	SVR	0.221	0.779	0.114
PIC	t4	RF	1.965	0.965	0.116
PIC	t4	XGBoost	0.823	0.177	0.097
PIC	t4	CNN	1.231	0.231	0.038
PIC	t4	RNN	0.402	0.598	0.367
PIC	t4	Single-EGT	0.386	0.614	0.294
PIC	t4	MTL-EGT	0.575	0.425	0.260
PIC	t5	GBLUP	1.181	0.181	0.398
PIC	t5	SVR	0.160	0.840	0.070
PIC	t5	RF	2.319	1.319	0.393
PIC	t5	XGBoost	0.859	0.141	0.391
PIC	t5	CNN	0.373	0.627	0.264
PIC	t5	RNN	0.201	0.799	0.387
PIC	t5	Single-EGT	0.296	0.704	0.166
PIC	t5	MTL-EGT	0.429	0.571	0.370

Note: Bias is the mean of (predicted − true), divided by the trait's phenotypic standard deviation estimated on the training dataset; slope is the regression of true phenotype on predicted value. Columns are, in order: dataset, trait, model, regression slope, the absolute deviation of that slope from unity, and prediction bias divided by the phenotypic standard deviation.

Table S3: Marker quality control applied to each dataset and the number of markers a MAF ≥ 0.01 and call rate ≥ 0.95 mask would remove.

Dataset	Retained markers	MAF filter	Call-rate filter	HWE filter	Markers failing the rule
PigHeaT	50735	yes	yes	none	35 (below MAF 0.01 inside the training split)
PIC	52843	no	no	none	2504
BloodLipid	61565	no	no	none	5413 (8.8%: 1608 call rate, 3883 MAF, 3126 monomorphic)

Note: Header columns are, in order: dataset, retained markers, MAF filter, call-rate filter, HWE filter, markers failing the rule. PigHeaT was assembled in this study and receives the in-house pipeline described in Section 2.1; the mask was computed on the full 1,148-animal cohort before splitting. PIC and BloodLipid are used as released by their repositories. No Hardy–Weinberg equilibrium filtering was applied to any dataset, because the pipeline implements no HWE test.

Table S4: Phenotype distributions of the retained traits on the training dataset.

Dataset	Trait	Training animals with a record	SD	Skewness	Integer-valued
PigHeaT	BFT19	804	2.036	0.79	no
PigHeaT	RT1923	798	0.229	0.23	no
PigHeaT	RFI	795	336.528	1.28	no
BloodLipid	HDL-C	847	0.336	0.84	no
BloodLipid	LDL-C	849	0.281	0.53	no
BloodLipid	TCHOL	851	0.354	0.29	no
BloodLipid	TG	849	0.172	1.39	no
PIC	t1	2210	1.170	1.26	no
PIC	t2	2080	1.092	0.08	no
PIC	t3	2112	1.015	0.03	no
PIC	t4	2124	2.274	0.55	no
PIC	t5	2155	60.255	0.25	no

Note: Columns after the trait name are: training-set size, standard deviation, skewness and whether the trait is integer-valued. The PigHeaT rows are the three traits reported in the study. Validation sizes are 122 for BFT19 and RT1923, 120 for RFI, 123 for the four BloodLipid traits, and 308, 283, 341, 340 and 341 for PIC t1 to t5; the exact test-set size of every trait is given in Table S1.

Table S5: Local linkage disequilibrium around MARC0013088 (SSC13:140,497,749, Sscrofa10.2).

Quantity	Value
Focal marker	MARC0013088
Assembly	Sscrofa10.2
Coordinate	SSC13:140497749
Animals in the pooled cohort	1259
Neighbour markers within ±1 Mb	40
Maximum r^2 to a neighbour	0.010958096448234396
Median r^2 to a neighbour	0.0029126915333651665
Neighbours with $r^2 > 0.2$	0
Minor allele frequency, pooled	0.028309409888357256

Note: Computed over all 1,259 genotyped animals and all markers within ±1 Mb. The two columns give the quantity and its value.

Table S6: Allele frequency of MARC0013088 by population.

Population	Animals	Minor-allele carriers	Minor allele frequency
DLY	610	49	0.040
Erhualian	333	0	0.000
Laiwu	316	22	0.035
Pooled	1259	71	0.028

Note: Columns are population, number of animals, minor-allele carriers and minor allele frequency.

Table S7: Effect of correcting the phenotypes for population structure on test-set PCC.

Trait	Variance explained by the ten PCs (%)	PCC before correction	PCC after correction
TG	17.6	0.620	0.280
LDL-C	9.9	0.236	0.296
TCHOL	6.2	0.298	0.175

Note: Each phenotype was residualised on the first ten principal components fitted on the training dataset only, and one EGT configuration was then retrained on the residualised phenotype with every other setting unchanged, so that the "before" and "after" columns differ only by the correction. The configuration and protocol are: single-trait EGT, 16 tokens of 64 dimensions, hybrid Pearson–MSE objective with $\alpha = 0.05$, dropout 0.1, 120 epochs, learning rate 5×10^{-4}, one training run (selection seed 42) chosen on the validation dataset. Because this is a single run without the five replicate seeds and without the seed-to-seed averaging used in the benchmark, a single number is reported here whereas Tables 2a and S1 of the main text report the mean ± SD over five replicates; the two are therefore not numerically interchangeable, and that is the reason the pre-correction values here (for example 0.620 against 0.595 ± 0.033 for TG, and 0.236 against 0.125 ± 0.020 for LDL-C) differ from the benchmark values for the same traits. The comparison within each row, before against after correction, is unaffected, and the pooled-cohort accuracies of TG and TCHOL do not represent within-population genetic prediction. Columns are, in order: trait, the share of phenotypic variance the ten principal components explain, and test-set PCC before and after the correction.

Table S8: Attribution of MARC0013088 before and after correcting for population structure.

Trait	Importance before correction	Importance after correction	Rank before	Rank after
LDL-C	0.137	0.162	1	1
TCHOL	0.639	0.141	1	1
TG	0.024	0.378	3	2

Note: Columns are trait, importance before correction, importance after correction, rank before and rank after. The attribution ranking is unchanged or improved by the correction.

Table S9: Comparison with the published genome-based GWAS results of Yang et al. (2015) for these animals.

Chromosome	Published observation	Comparison with this study	Verdict
SSC13	1 suggestive SNP (ASGA0058243, 84,662,548 bp, HDL-C/LDL-C ratio in DLY, $p = 1.8 \times 10^{-5}$, $q = 0.239$)	55.8 Mb from MARC0013088	does not survive correction
SSC13	MARC0013088	absent from both supplementary tables	power-limited: the marker is monomorphic in Erhualian and carried by only 71 animals cohort-wide
SSC3	106 of the 122 suggestive SNPs, spanning 116.4–143.8 Mb	strongest signal H3GA0010460 (LDL-C in Laiwu, $q = 0.003$) inside the *APOB* window	–
SSC3	model attribution at ∼121.75 Mb (LDL-C importance 0.107, TCHOL 0.514)	within about 3 Mb of the reported peak, in a region covered by 80 array markers	partial correspondence

Note: Rows are, in order: chromosome, published observation, comparison with this study, and verdict. The published observations were extracted from Tables S4 (suggestive SNPs in the three populations, 122 rows) and S5 (five-population meta-analysis, 51 rows) of Yang et al. (2015).

Table S10: Animal QTLdb records overlapping the SSC13 candidate region (43 records).

QTL_ID	Reported trait	Breeds	Map location
25	Backfat at tenth rib	—	5 (0-6.2) (cM)
70	Average backfat thickness	—	27 (9.17-9.50) (cM)
71	Backfat at tenth rib	—	23 (8.07-8.18) (cM)
72	backfat at last rib	—	36 (13.48-13.59) (cM)
286	Average backfat thickness	—	70.1 (64.11-64.51) (cM)
287	Backfat at first rib	—	70.1 (64.11-64.51) (cM)
288	Backfat at last lumbar	—	70.1 (64.11-64.51) (cM)
652	Average backfat thickness	—	14.1 (0-35.4) (cM)
1132	Backfat weight	—	43.1 (27.9-62.2) (cM)
1134	External fat on ham	—	98.1 (74.4-101.6) (cM)
1135	backfat at last rib	—	97.2 (74.4-101.6) (cM)
1137	Shoulder external fat weight	—	108.0 (101.6-121.2) (cM)
1140	External fat on ham	—	96.1 (74.4-101.6) (cM)
1141	Fat-cuts percentage	—	45.6 (43.1-53) (cM)
3087	Side fat thickness	—	69 (61-79.3) (cM)
3672	backfat at last rib	—	96.65 (62.2-79.3) (cM)
3968	Ham fat thickness	—	84.47 (58-79.3) (cM)
4163	backfat at P2 position	—	96.65 (62.2-79.3) (cM)
5583	Intramuscular fat content	finnish landrace (LBO:0000361); Hampshire (LBO:0000366)	30 (10.45-10.54) (cM)
5718	Average backfat thickness	duroc (LBO:0000358); pietrain (LBO:0000395)	30.1 (1.6-35.4) (cM)
5719	Backfat at tenth rib	duroc (LBO:0000358); pietrain (LBO:0000395)	32.3 (1.6-35.4) (cM)
5720	Fat area	duroc (LBO:0000358); pietrain (LBO:0000395)	53.3 (35.4-79.3) (cM)
7379	Intramuscular fat content	duroc (LBO:0000358); Berkshire (LBO:0000348)	78.3 (84.45-84.60) (cM)
7404	Intramuscular fat content	duroc (LBO:0000358); large white (LBO:0000378)	52 (29.88-30.15) (cM)
7437	Monounsaturated fatty acid content	duroc (LBO:0000358); large white (LBO:0000378)	45 (21.03-21.27) (cM)
7700	HDL cholesterol	duroc (LBO:0000358)	79.6 (85.60-86.04) (cM)
7701	HDL/LDL ratio	duroc (LBO:0000358)	79.6 (85.60-86.04) (cM)
7707	LDL cholesterol	duroc (LBO:0000358)	76.6 (82.09-82.34) (cM)
7708	HDL/LDL ratio	duroc (LBO:0000358)	74.6 (79.01-79.34) (cM)
7712	LDL cholesterol	duroc (LBO:0000358)	105.6 (117.07-117.14) (cM)
7724	Triglyceride level	duroc (LBO:0000358)	6.6 (3.50-3.58) (cM)
7726	Cholesterol level	duroc (LBO:0000358)	85.6 (93.95-94.10) (cM)
7730	Cholesterol level	duroc (LBO:0000358)	73.6 (75.31-75.75) (cM)
15077	Cholesterol level	—	105 (77.1-121.2) (cM)
15078	Triglyceride level	—	20 (14.1-121.2) (cM)
16840	backfat at last rib	meishan (LBO:0000381); pietrain (LBO:0000395); Wild boar (LBO:0000695)	39.5 (27.9-56.8) (cM)
16984	Intramuscular fat content	duroc (LBO:0000358)	57 (40.06-40.41) (cM)
17725	LDL cholesterol	duroc (LBO:0000358); pietrain (LBO:0000395)	59.63 (1.6-79.3) (cM)
17784	backfat at last rib	iberian (LBO:0000369); meishan (LBO:0000381)	67.3 (53-79.3) (cM)
17785	backfat at last rib	iberian (LBO:0000369); meishan (LBO:0000381)	79.3 (85.35-85.59) (cM)
18641	Fat area percentage in carcass	duroc (LBO:0000358)	56.25 (53-62.2) (cM)
21265	intestinal fat weight	duroc (LBO:0000358); Erhualian (LBO:0000691)	50.3 (31.3 - 67.4) (cM)
21404	backfat at last rib	duroc (LBO:0000358); pietrain (LBO:0000395)	101.6 (79.3-102.2) (cM)

Note: Records are from the database's permanent deposition of its retired linkage-map-based QTL, positioned in centimorgans on the porcine linkage map of Rohrer et al. (Rohrer, G.A.; Alexander, L.J.; Hu, Z.; Smith, T.P.; Keele, J.W.; Beattie, C.W. A comprehensive map of the porcine genome. *Genome Res.* **1996**, *6*, 371–391, https://doi.org/10.1101/gr.6.5.371); the live overlap query was unavailable at the time of analysis, and the deposition does not include the genome-based QTL and SNP-association records. Columns: QTL_ID, Reported trait, Breeds, Map location. A dash marks a record for which no breed is recorded.

Table S11: Repeated masking of the top-attributed bins against a marker-count-matched random control.

Trait	Masking fraction	Top-bin ΔPCC	Matched-control mean	Matched-control interval	Draws	ΔPCC below control interval
HDL-C	0.01	-0.0052	-0.0006	[-0.0041, +0.0019]	20	yes
HDL-C	0.05	-0.0127	+0.0006	[-0.0049, +0.0055]	20	yes
HDL-C	0.10	-0.0169	-0.0011	[-0.0039, +0.0020]	20	yes
LDL-C	0.01	+0.0102	+0.0004	[-0.0080, +0.0089]	20	no
LDL-C	0.05	-0.0065	+0.0014	[-0.0103, +0.0121]	20	no
LDL-C	0.10	-0.0231	+0.0036	[-0.0079, +0.0160]	20	yes
TCHOL	0.01	+0.0034	-0.0012	[-0.0045, +0.0015]	20	no
TCHOL	0.05	+0.0135	-0.0016	[-0.0073, +0.0036]	20	no
TCHOL	0.10	+0.0268	-0.0015	[-0.0065, +0.0070]	20	no
TG	0.01	-0.0040	-0.0028	[-0.0170, +0.0186]	20	no
TG	0.05	-0.0313	+0.0000	[-0.0206, +0.0181]	20	yes
TG	0.10	-0.0477	-0.0029	[-0.0267, +0.0191]	20	yes

Note: Twenty independent random draws per trait and fraction; the interval is the 2.5–97.5 percentile interval of those draws, and the matched control masks the same number of markers as the top-attributed bins. A negative Δ PCC means that masking the top-ranked bins reduced test-set PCC. Columns are trait, masking fraction, top-bin ΔPCC, matched-control mean, matched-control interval, number of draws, and whether the top-bin ΔPCC lies below the control interval. The top-bin ΔPCC falls below the control interval in six of the twelve trait–fraction combinations: HDL-C at every fraction, LDL-C at 10%, and TG at 5% and 10%. For LDL-C at 1% and for TCHOL at all three fractions the shift is positive and lies above the control interval, and the two remaining combinations (LDL-C at 5% and TG at 1%) lie inside it.

Table S12: Locus-scale controls for the masking analysis.

Region masked	LDL-C ΔPCC	TCHOL ΔPCC	TG ΔPCC
SSC3 whole chrom	-0.0174	-0.0193	-0.0067
APOB locus 5Mb	-0.0029	-0.0020	-0.0018
PCSK9 locus 5Mb	+0.0003	+0.0008	-0.0088
SSC13 whole chrom	-0.0077	+0.0049	-0.0067
SSC13 candidate	-0.0009	+0.0002	+0.0002
MARC0013088 window	-0.0011	-0.0002	-0.0010

Note: SSC3, which carries the published *APOB* signal for these animals, is the positive control; the SSC13 regions are the negative controls, at chromosome, candidate-region and single-marker scale. Masking SSC3 is the most damaging single region for LDL-C and TCHOL, whereas every SSC13 row is inert: the whole chromosome moves test PCC by at most 0.008 and the candidate region and the focal marker's own window by at most 0.0011, so the attributed SSC13 region carries attribution but no measurable predictive dependence. Columns are, in order: the masked region and the resulting change in test-set PCC for each of the three traits.

Table S13: Complete BloodLipid loss-weight grid.

Score term	MSE weight (α)	Score weight ($1-\alpha$)	Mean PCC	Mean RMSE	Mean MAE
Pearson	0.00	1.00	0.289	0.259	0.213
Pearson	0.05	0.95	0.293	0.253	0.199
Pearson	0.10	0.90	0.274	0.264	0.207
Pearson	0.15	0.85	0.258	0.277	0.219
Pearson	0.25	0.75	0.255	0.286	0.224
Pearson	0.50	0.50	0.291	0.269	0.210
Pearson	0.75	0.25	0.290	0.264	0.206
Pearson	1.00	0.00	0.291	0.267	0.208
R^2	0.05	0.95	0.277	0.262	0.206
R^2	0.10	0.90	0.277	0.263	0.206
R^2	0.15	0.85	0.272	0.263	0.206
R^2	0.25	0.75	0.280	0.268	0.208
R^2	0.50	0.50	0.276	0.269	0.210
R^2	0.75	0.25	0.279	0.266	0.208
Spearman	0.05	0.95	0.256	0.277	0.219
Spearman	0.10	0.90	0.259	0.275	0.215
Spearman	0.15	0.85	0.259	0.282	0.219
Spearman	0.25	0.75	0.275	0.272	0.214
Spearman	0.50	0.50	0.266	0.284	0.222
Spearman	0.75	0.25	0.263	0.289	0.228

Note: Values are means over the four BloodLipid traits; the setting used for the reported models is $\alpha = 0.05$ with a Pearson score term. Columns are, in order: the score term, the MSE and score weights, and the mean PCC, RMSE and MAE over the four BloodLipid traits.

Table S14: Optimisation settings for every model.

Model	Optimiser	Learning rate	Weight decay	Schedule	Warmup	Gradient clipping	Mixed precision	Early-stopping patience
EGT (single-trait and MTL)	AdamW	5e-4 or 1e-3 by dataset	1e-4	cosine annealing over the requested epochs	no	5.0	yes	25
CNN and RNN baselines	AdamW	1e-3 (tuned on validation)	1e-4	none	no	none	no	15
Autoencoder	AdamW	configured value	1e-5	cosine annealing over 30 epochs	no	none	no	8
GBLUP	closed-form mixed-model solution	not applicable	not applicable	not applicable	not applicable	not applicable	no	not applicable
SVR	linear kernel, prior setting	C = 1.0, epsilon = 0.1	not applicable	not applicable	not applicable	not applicable	no	not applicable
Random forest	ensemble, tuned on validation	500 trees, max_features sqrt	not applicable	not applicable	not applicable	not applicable	no	not applicable
XGBoost	boosted ensemble, prior setting	0.03 with 800 trees, depth 4	not applicable	not applicable	not applicable	not applicable	no	not applicable

Note: The values listed here are the settings used for every reported result. Hyperparameters were tuned on the validation dataset for every model, the baselines included: the EGT learning rate and the autoencoder settings were selected over the search grids of the tuning campaign, and the random forest, CNN and RNN had their optimiser settings and learning rate searched over a candidate set, selected on the validation dataset with the test datasets untouched. Support-vector regression and XGBoost use settings chosen from prior knowledge of high-dimensional genomic prediction rather than by search, which is why they are labelled as prior settings here. GBLUP has no iterative optimiser, its variance components being estimated by REML. The tuning effort is therefore not identical across models, a difference of range rather than of principle, and it is stated as a limitation in Section 2.3 of the main text. Columns are, in order: model, optimiser, learning rate, weight decay, schedule, warmup, gradient clipping, mixed precision and early-stopping patience.

Table S15: The reported comparison under two selection rules. Under Rule A each side is taken at its best on the test dataset, the convention of Table 2 of the main text. Under Rule B each side is taken at its best on the validation dataset, so no test-set performance enters the choice of which model is reported; the test dataset is then read once, for the models so selected. Both sides of every comparison are listed, with their test-set PCC.

		**Rule A (test-best)**				**Rule B (validation-best)**			
**Dataset**	**Trait**	**EGT**	**PCC**	**Baseline**	**Δ**	**EGT**	**PCC**	**Baseline**	**Δ**
PigHeaT	BFT19	Single-EGT	0.549	GBLUP	+0.035	Single-EGT	0.549	GBLUP	+0.035
PigHeaT	RT1923	Single-EGT	0.149	RF	+0.007	Single-EGT	0.149	GBLUP	+0.035
PigHeaT	RFI	Single-EGT	0.179	GBLUP	+0.006	Single-EGT	0.179	GBLUP	+0.006
BloodLipid	HDL-C	MTL-EGT	0.085	SVR	+0.025	MTL-EGT	0.085	GBLUP	+0.031
BloodLipid	LDL-C	MTL-EGT	0.181	XGBoost	+0.057	Single-EGT	0.125	XGBoost	+0.001
BloodLipid	TCHOL	Single-EGT	0.309	SVR	+0.015	Single-EGT	0.309	CNN	+0.200
BloodLipid	TG	Single-EGT	0.595	GBLUP	+0.041	Single-EGT	0.595	CNN	+0.093
PIC	t1	MTL-EGT	0.046	CNN	−0.015	Single-EGT	0.044	CNN	−0.017
PIC	t2	MTL-EGT	0.091	RF	−0.029	Single-EGT	0.079	GBLUP	+0.027
PIC	t3	MTL-EGT	0.075	RF	+0.032	MTL-EGT	0.075	GBLUP	+0.062
PIC	t4	MTL-EGT	0.345	GBLUP	+0.037	Single-EGT	0.342	GBLUP	+0.034
PIC	t5	MTL-EGT	0.183	GBLUP	+0.034	Single-EGT	0.173	GBLUP	+0.024

Note: The **EGT** column names the reported EGT variant and the **Baseline** column the reported conventional model, for the rule in question. **PCC** is the test-set PCC of the EGT variant named in the same row, and Δ is that value minus the test-set PCC of the conventional model named in the same row; Δ is positive where EGT is ahead. The two rules choose a different EGT variant in five of the twelve traits (LDL-C, t1, t2, t4 and t5) and a different conventional model in six (RT1923, HDL-C, TCHOL, TG, t2 and t3). EGT is ahead in ten of the twelve traits under Rule A and in eleven under Rule B; the one direction that changes is PIC t2, where the Rule A margin of −0.029 becomes +0.027. The largest reduction is BloodLipid LDL-C, where the Rule A margin of +0.057 falls to +0.001 because the validation dataset prefers single-trait EGT (0.344 against 0.290) while the test dataset prefers MTL-EGT. Under both rules the model that is reported is fixed before the test dataset is read.

Table S16: Validation-set PCC of every model for the twelve reported traits.

**Dataset**	**Trait**	**GBLUP**	**SVR**	**RF**	**XGBoost**	**CNN**	**RNN**	**Single-EGT**	**MTL-EGT**
PigHeaT	BFT19	0.412	0.364	0.318	0.397	0.033	0.286	0.420	0.405
PigHeaT	RT1923	0.301	0.171	0.249	0.293	0.197	0.143	0.292	0.225
PigHeaT	RFI	0.117	0.081	0.042	0.023	0.006	0.003	0.145	0.054
BloodLipid	HDL-C	0.106	0.023	0.067	0.083	0.060	0.074	0.127	0.130
BloodLipid	LDL-C	0.340	0.302	0.371	0.413	0.330	0.308	0.344	0.290
BloodLipid	TCHOL	0.065	0.083	0.077	0.090	0.239	0.129	0.168	0.131
BloodLipid	TG	0.544	0.398	0.533	0.465	0.546	0.462	0.503	0.439
PIC	t1	0.060	0.067	0.065	0.058	0.094	0.080	0.105	0.068
PIC	t2	0.257	0.131	0.215	0.186	0.113	0.159	0.239	0.220
PIC	t3	0.262	0.112	0.231	0.152	0.134	0.172	0.192	0.195
PIC	t4	0.427	0.275	0.386	0.362	0.345	0.353	0.362	0.361
PIC	t5	0.339	0.171	0.206	0.302	0.235	0.256	0.329	0.292

Note: PCC of each of the eight models on the validation dataset of each reported trait. These are the values on which Rule B of Table S15 is based, and they are given here so that the selection of the reported model can be checked directly against the test-set values of Table S1. The test-set counterpart of this table is Table S1.
